# Neuromuscular study of early branching *Diuronotus aspetos* (Paucitubulatina) yields insights into the evolution of organs systems in Gastrotricha

**DOI:** 10.1186/s40851-016-0054-3

**Published:** 2016-09-22

**Authors:** Nicolas Bekkouche, Katrine Worsaae

**Affiliations:** Marine Biological Section, Department of Biology, University of Copenhagen, Universitetsparken 4, 2100 Copenhagen Ø, Denmark

**Keywords:** Neurobiology, Nervous system, Musculature, Ciliary structures, Meiofauna, Chaetonotida, Spiralia, Molecular phylogeny

## Abstract

**Background:**

*Diuronotus* is one of the most recently described genera of Paucitubulatina, one of the three major clades in Gastrotricha. Its morphology suggests that *Diuronotus* is an early branch of Paucitubulatina, making it a key taxon for understanding the evolution of this morphologically understudied group. Here we test its phylogenetic position employing molecular data, and provide detailed descriptions of the muscular, nervous, and ciliary systems of *Diuronotus aspetos*, using immunohistochemistry and confocal laser scanning microscopy.

**Results:**

We confirm the proposed position of *D. aspetos* within Muselliferidae, and find this family to be the sister group to Xenotrichulidae. The muscular system, revealed by F-actin staining, shows a simple, but unique organization of the trunk musculature with a reduction to three pairs of longitudinal muscles and addition of up to five pairs of dorso-ventral muscles, versus the six longitudinal and two dorso-ventral pairs found in most Paucitubulatina. Using acetylated α-tubulin immunoreactivity, we describe the pharynx in detail, including new nervous structures, two pairs of sensory cilia, and a unique canal system. The central nervous system, as revealed by immunohistochemistry, shows the general pattern of Gastrotricha having a bilobed brain and a pair of ventro-longitudinal nerve cords. However, in addition are found an anterior nerve ring, several anterior longitudinal nerves, and four ventral commissures (pharyngeal, trunk, pre-anal, and terminal). Two pairs of protonephridia are documented, while other Paucitubulatina have one. Moreover, the precise arrangement of multiciliated cells is unraveled, yielding a pattern of possibly systematic importance.

**Conclusion:**

Several neural structures of *Diuronotus* resemble those found in *Xenotrichula* (Xenotrichulidae) and may constitute new apomorphies of Paucitubulatina, or even Gastrotricha. In order to test these new evolutionary hypotheses, comparable morphological data from other understudied gastrotrich branches and a better resolution of the basal nodes of the gastrotrich phylogeny are warranted. Nonetheless, the present study offers new insights into the evolution of organ systems and systematic importance of so-far neglected characters in Gastrotricha.

## Background

Gastrotricha are small, often sub-millimetre, interstitial worms, ubiquitously found in most aquatic environments with a long debated phylogenetic position [1–3]. They were first considered closely related to various meiofaunal, protostome groups, such as rotifers (Trochelminthes [4]), kinorhynchs (Nematorhyncha [5]) or Gnathostomulida (Neotrichozoa [6, 7]). Later, molecular phylogenies placed them within Spiralia with uncertain affinities; within the debated group Platyzoa, comprising Gastrotricha, Platyhelminthes and Gnathifera [1, 8, 9]. Recent phylogenomic studies propose a sister group relationship between Platyhelminthes and Gastrotricha [3, 10]. However, the controversy over the phylogenetic position of Gastrotricha masks other problems within the group. Indeed, compared to the diversity and omnipresence of these animals, relatively few phylogenetic and detailed morphological studies have been conducted on this group and the evolution of, e.g., nervous system, muscular system and nephridia is unresolved [11–13]. Its diversity also remains largely unexplored, as exemplified by the recent erection of the family Hummondasyidae (Macrodasyida) in 2014 [14] and the genera *Thaidasys* in 2015 [15] and *Bifidochaetus* in 2016 [16].

*Diuronotus* [17] is another recently described gastrotrich genus (2005), comprising two described species: *Diuronotus aspetos* Todaro et al. [17] and, *Diuronotus rupperti* Todaro et al. [17], and one undescribed species *Diuronotus* sp. [18–20], transferred from *Halichaetonotus* [17]. These are all found in marine interstitial environments of the North Atlantic; *D. aspetos* from Greenland [17] and Germany [2, 21], *D. rupperti* from Denmark [17] and *Diuronotus* sp. from North Carolina, USA [18]. *Diuronotus* was placed in Muselliferidae (Paucitubulatina, Chaetonitida) next to *Musellifer* [22] with which it shares the presence of a ciliated so-called ‘muzzle’ (or snout) and specific ultrastructural traits of scales and sperm [23].

Gastrotricha are divided into two main taxa: the supposedly monophyletic Macrodasyida and the possibly paraphyletic Chaetonotida, divided further into the Multitubulatina, (consisting of one genus, *Neodasys*, and possessing multiple adhesive glands) and the diverse Paucitubulatina (possessing generally only two adhesive tubes) [24]. Muselliferidae, belonging to Paucitubulatina, is the possible sister group to all remaining Paucitubulatina according to morphological [22, 25] and molecular [26, 27] studies. However, Paps and Riutorts [28] propose an alternative topology in which Xenotrichulidae is positioned as sister group of the remaining Paucitubulatina, and Muselliferidae being the sister group to Chaetonotidae. Kieneke et al. [29] find Proichthydiidae as sister group to the remaining Paucitubulatina, with Muselliferidae forming a clade together with Xenotrichulidae sister group to other Paucitubulatina. These different topologies overall suggest a key position of Muselliferidae within Gastrotricha, emphasizing the importance of this family for understanding the evolution of Gastrotricha. Indeed, some features of Muselliferidae, namely its marine habitat and well-developed hermaphroditism, are thought to be plesiomorphic character traits of Chaetonotida. However, detailed morphological studies on this family are lacking, most likely due to the paucity of these animals and their late discovery [26, 30].

Recently, a series of papers employing confocal laser scanning microscopy (CLSM) described the detailed muscular arrangement of several Paucitubulatina, namely *Musellifer* [22], Xenotrichulidae [22, 31], Chaetonotidae [22, 32], and Dasydytidae [11, 33], and notably, the helicoidal musculature, proposed to be a gastrotrich synapomorphy [34]. These recent reports were used to infer the plesiomorphic arrangement of the musculature of Gastrotricha as constituted by two ventro-lateral longitudinal muscles surrounded by outer circular muscles, and longitudinal splanchnic muscles surrounded by helicoidal and intestinal circular muscles [2]. In Paucitubulatina, the longitudinal muscles appear to be more numerous, and the outer circular muscles, if present, are incomplete and consist of dorso-ventral muscles [2]. These dorso-ventral or semi-circular muscles are found in marine chaetonotids [22], but are often missing or highly reduced in freshwater chaetonotids [11, 22, 33] emphasizing the importance of studying the marine *Diuronotus* in order to resolve their evolution and contribute to the broader understanding of muscular evolution within Gastrotricha.

To date, only one confocal study on *Xenotrichula* has described the nervous system of a member of Paucitubulatina in detail [12], while it has been extensively described for Multitubulatina (*Neodasys*) [13] and in several Macrodasyida with combined immunohistochemistry and CLSM (e.g., [35, 36]), or transmission electron microscopy (TEM) [37]. One of the conclusions of the *Xenotrichula* study [13] is the low structural variation of the nervous system within Gastrotricha, which always comprises a bilobed brain with a ventral commissure, a pair of anteriorly projecting longitudinal nerves, a pair of ventro-lateral nerve cords along the trunk, and a terminal commissure. These features were also interpreted as ancestral conditions of Gastrotricha in Kieneke and Schmidt-Rhaesa (2015) [2, 12]. Yet only a single Paucitubulatina, *Xenotrichula*, was considered for this state reconstruction. Moreover, substantial variation exists, such as the presence of an additional ventral nerve in *Oregodasys cirratus* Rothe & Schmidt-Rhaesa [38] and dorsal nerves in *Xenodasys riedli* (Schöpfer-Sterrer, 1969) [39, 40], or additional trunk commissures in *Dactylopodola* and *Oregodasys cirratus.* These studies highlight the unexplored diversity of gastrotrich nervous systems, which may be especially relevant in the diverse group of Paucitubulatina, in which only one study of the nervous system has been reported so far [13] and given the total lack of data on Muselliferidae.

Several studies have described the ultrastructure and repartition of protonephridia in Gastrotricha, with a few of them addressing species of Paucitubulatina [41, 42]. It has been suggested that members of Paucitubulatina always possess one pair of trunk protonephridia [41], although again, data on Muselliferidae are lacking. Each nephridium was found to encompass two monociliated terminal cells with coaxial cilia, a long canal cell, and a nephridopore cell [2].

In order to enhance our understanding of the evolution of major organ systems within Gastrotricha we acquired new morphological data on *Diuronotus aspetos*, using CLSM techniques and immunohistochemistry to describe the detailed arrangement of the musculature, nervous system, and ciliation. To assess the previously proposed relationship of *D. aspetos* within Muselliferidae, we analyzed the phylogenetic position within Chaetonotida, using molecular data. In this phylogenetic context, the morphology of *Diuronotus* is compared and discussed relative to other Chaetonotida, and Gastrotricha in general, leading to the proposition of several new homologies.

## Methods

### Collection

For *Diuronotus aspetos*, the samples were taken with a mini van Veen grab from shallow water (3–6 m water depth) of Flakkerhuk (69°38.63’N 51°51.13’W), Disko Island, West Greenland. All specimens were collected during the Arctic summer in August 2013. Sediment was well-sorted sand of fine to medium grain size. The specimens have been extracted with MgCl_2_ narcotization and decantation.

For DNA, specimens of *Xenotrichula* sp. have been sampled in Ystad, Sweden (55°26.28’N 13°55.44’E) in subterranean environments on a beach with fine to medium-sized sand, and extracted with MgCl_2_ narcotization and decantation. Marine *Aspidiophorus* sp. were sampled from cultures of *Dinophilus gyrociliatus* from Copenhagen University, where they are contaminants and unfortunately of unknown origin.

### Sequence acquisition

Total genomic DNA was obtained from whole specimens using the Qiagen DNeasy Blood & Tissue Kit (Qiagen Inc., Valencia, CA, USA) following the manufacturer’s protocol, except for performing the DNA elution in 160 μL of AE buffer in order to increase the final DNA concentration.

Polymerase chain reactions (PCR) using Sanger based markers were prepared to a final volume of 25 μL with 12.5 μL of GoTaq® Green Master Mix (Promega Corporation, Madison, WI, USA), 1 μL of each primer (10 μM concentration), 10–8.5 μL of Milli-Q water (adjusted to amount of DNA template), and 0.5–2 μL of DNA template. Reaction mixtures were heated in a Bio-Rad G1000 Thermal Cycler at 94 °C for three minutes, followed by 35–40 cycles (primer specific) of 94 °C for 30 s, specific primer pair annealing temperatures for 30 s, and an extension at 68 °C for 45 s (unless indicated otherwise), and a final extension phase of 5 min at 72 °C. The COI primer set dgLCO1490/dgHCO2198 [43] was run with two cycling steps, both variable in temperature. COI annealing temperatures were 45 °C for 45 s and 51 °C for 45 s, respectively with extensions of 30s. Overlapping fragments of the small 18S rDNA (ca. 1800 bp) were obtained using paired primers corresponding to fragments 1 and 3 of the 18S rDNA [44]: (1) 18S1f/18S5R (ca. 900 bp) and (3) 18Sa2.0/18S9r (ca. 800 bp) both overlapping. Both primer sets (1) and (3) had annealing temperatures set to 49 °C. The 28S primer set used was 28SD3/28SG758 [45, 46] with an annealing temperature of 53 °C.

All newly generated and integrated sequences were deposited in the GenBank ® database with the following accession numbers KX531001 to KX531007 (Table 1).

**Table 1:** Sequences used for the phylogenetic reconstruction with their corresponding GenBank accession number. See material and methods for further information on the acquisitions of the sequences of *Diuronotus aspetos*

Species name	18S	28S	COI
*Arenotus strixinoi*	JQ798537.1	JQ798608.1	JQ798677.1
*Aspidiophorus* kw654	KX531002	KX531001	No
*Aspidiophorus ophiodermus*	JN185463.1	JN185510.1	JN185544.1
*Aspidiophorus paramediterraneus*	JQ798538.1	JQ798609.1	JQ798678.1
*Aspidiophorus polystictos* TK76	JQ798598.1	JQ798665.1	JQ798727.1
*Aspidiophorus polystictos* TK75	JQ798597.1	JQ798664.1	JQ798726.1
*Aspidiophorus* sp.3	JQ798559.1	JQ798629.1	JQ798694.1
*Aspidiophorus tentaculatus* TK120	JQ798553.1	JQ798625.1	JQ798690.1
*Aspidiophorus tentaculatus* TK228	JQ798591.1	JQ798659.1	JQ798721.1
*Aspidiophorus tetrachaetus*	JN185505.1	JN185540.1	JN185576.1
*Chaetonotus laroides*	JQ798580.1	No	JQ798712.1
*Chaetonotus* cf. *sphagnophilus*	JQ798604.1	JQ798671.1	JQ798733.1
*Chaetonotus* cf. *dispar*	JQ798561.1	JQ798631.1	JQ798696.1
*Chaetonotus* cf. *hystrix*	JQ798603.1	Q798670.1	JQ798732.1
*Chaetonotus* cf. *laroides* TK86	JQ798602.1	JQ798669.1	JQ798731.1
*Chaetonotus* cf. *maximus* TK186	JQ798574.1	JQ798646.1	JQ798706.1
*Chaetonotus heterocanthus* TK100	JQ798543.1	JQ798615.1	JQ798681.1
*Chaetonotus mariae*	JQ798558.1	JQ798628.1	No
*Chaetonotus neptuni* MT61	JQ798539.1	JQ798610.1	JQ798679.1
*Chaetonotus uncinus*	JQ798540.1	JQ798611.1	No
*Chaetonotus* cf. *novenarius*	JQ798566.1	JQ798636.1	JQ798699.1
*Dactylopodola mesotyphle*	JF357651.1	JF357699.1	JF432036.1
*Dasydytes papaveroi* TK157	JQ798571.1	JQ798640.1	JQ798703.1
*Diuronotus aspetos*	KX531005 and SRX1121926	KX531006 or SRX1121926	KX531007 or SRX1121926
*Draculiciteria tesellata* MT63	JN185457.1	JN185506.1	JN185541.1
*Draculiciteria tesellata* TK142	JN185470.1	JN185516.1	JN185549.1
*Halichaetonotus aculifer*	JQ798550.1	JQ798622.1	JQ798688.1
*Halichaetonotus euromarinus*	JQ798551.1	JQ798623.1	No
*Haltidytes squamosus*	JQ798567.1	JQ798637.1	No
*Heterolepidoderma macrops*	JN185469.1	JN185515.1	JN185548.1
*Heterolepidoderma* sp.2	JN185485.1	JQ798644.1	JN185563.1
*Heteroxenotrichula squamosa*	JQ798542.1	JQ798613.1	No
*Ichthydium skandicum* TK182	JQ798573.1	JQ798645.1	JQ798705.1
*Ichthydium squamigerum*	JQ798607.1	JQ798674.1	JQ798736.1
*Kijanebalola devestiva* TK240	KR822112.1	KR822117.1	KR822120.1
*Lepidochaetus brasilense* TK223	JN185495.1	JQ798658.1	JN185568.1
*Lepidochaetus zelinkai* TK94	JN185503.1	JN185538.1	JN185574.1
*Lepidodermella squamata* TK97	JN185504.1	JN185539.1	JN185575.1
*Macrodasys* sp.1	JF357654.1	JF357702.1	JF432040.1
*Megadasys* sp.1	JF357656.1	JF357704.1	JF432042.1
*Musellifer delamarei*	AM231775.1	No	No
*Musellifer reichardti*	KF578503.1	No	No
*Neodasys chaetonotoideus*	JQ798535.1	No	JQ798675.1
*Neodasys uchidai*	JQ798536.1	No	JQ798676.1
*Neogossea acanthocolla*	KR822114.1	KR822119.1	KR822121
*Neogossea antennigera* TK232	KR822110.1	KR822115.1	No
*Ornamentula paraensis* TK147	JQ798562.1	JQ798632.1	JQ798697.1
*Polymerurus nodicaudus* TK16*5*	JN185502.1	JN185537.1	JN185573.1
*Polymerurus rhomboides* TK217	JN185493.1	JN185533.1	JN185567.1
*Stylochaeta fusiformis*	JN185471.1	JN185517.1	JN185550.1
*Xenotrichula cf. intermedia*	JN185461.1	No	No
*Xenotrichula intermedia* MT71	JF357664.1	JF357712.1	No
*Xenotrichula* sp. kw655	KX531003	KX531004	No
*Xenotrichula punctata*	JN185464.1	JN185511.1	No
*Xenotrichula* sp*.* TK121	JF970234.1	No	No
*Xenotrichula* sp.1	JN185466.1	No	JN185545.1
*Xenotrichula velox* TK202	JN185488.1	JQ798652	No
*Xenotrichula velox* TK43	JN185499.1	No	No

### Phylogenetic analysis

Sequences were cleaned on BioEdit [47], and a consensus has been realized from the reverse and forward sequences. Sequences were blasted on NCBI [48]. In parallel, COI, 18S and 28S of *Diuronotus aspetos* were found from its transcriptome [3], using Blastall from NCBI. Sangers and transcriptome acquired COI, 18S and 28S genes were aligned and compared, showing low quality and short length of Sangers sequences. Consequently, COI and 28S of the transcriptome were kept, while a consensus of 18S from the transcriptome and the Sangers sequencing was done having an identical overlapping segment. This hybrid approach was possible since the specimens used for the transcriptome and the Sanger sequences came from the same sample. Sequences of *Aspidiophorus* sp. and *Xenotrichula* sp. were added to the dataset. Sequences of other gastrotrichs acquired from GenBank, based on the tree proposed by Kånneby et al. 2012 [49] were added, selecting sequences from each genus (except *Bifidochaetus* [16]) for which sequences were not available at the time of the analysis), and representing the shortest and deepest branches possible. Sequences from Kånneby et al. [26] for *Musellifer*, Kånneby and Todaro [50] for Neogosseidae, and Todaro et al. [51] for the macrodasyidans, outgroups have additionally been collected from GenBank. Subsequently, the sequences were aligned gene per gene with Muscle in Seaview [47], checked by hand, and the three genes were concatenated with Sequence Matrix [52]. Finally, this dataset was analyzed with Bayesian inference in MrBayes 3.2.6 [53] under the model GTR + I + Γ. The gamma shape parameter, the substitution rates, the proportion of invariable sites, and the character state frequencies were all unlinked. The dataset was partitioned according to each gene and by codon position for COI and analyzed with four MCMC chains for each run, for 30,000,000 generations. Chains were sampled every 1000^th^ generations and the burn-in was set to 25 %. Convergence of the two runs as well as analysis quality was ascertained by checking the log likelihood graphs, the average standard deviation of split frequencies, and the model fit with Tracer [54].

### Immunohistochemistry and CLSM

Specimens were anesthetized with isotonic magnesium chloride and fixed in 3.7 % paraformaldehyde in phosphate buffered saline (PBS) for 1 to 2 h at room temperature (RT), followed by six rinses in PBS and storage in PBS with 0.05 % NaN_3_. Triple or quadruple stainings were performed for the investigation of muscular, nervous, glandular and ciliary systems, including F-actin staining (Alexa Fluor 488-labelled phalloidin, INVITROGEN, Carlsbad, USA), DNA-staining (405 nm fluorescent DAPI) and antibodies against neurotransmitters and tubulinergic elements (monoclonal mouse anti-acetylated α-tubulin (SIGMA T6793, St. Louis, USA), polyclonal chicken anti acetylated α-tubulin (SAB3500023-100UG), polyclonal rabbit anti-serotonin (5-HT, SIGMA S5545) and anti-FMRF-amide (IMMUNOSTAR 20091, Hudson, USA)). Prior to adding the primary antibody-mix, the samples were pre-incubated with 1 % PTA (PBS + 1 % Triton-X, 0.05 % NaN3, 0.25 % BSA, and 5 % sucrose) for 1 h. Samples were incubated over night at RT in primary antibodies mixed 1:1 with glycerol (in a final 1:400 concentration). Subsequently, specimens were rinsed in PBS six times and incubated with the secondary antibodies conjugated with fluorochromes over night at RT (mixed 1:1 with glycerol; 1:400 goat anti-mouse labeled with CY5 (JACKSON IMMUNO-RESEARCH, West Grove, USA, 115-175-062), 1:400 goat anti-mouse labeled with TRITC (JACKSON IMMUNO-RESEARCH, West Grove, USA, 115-175-062), 1:400 goat anti-rabbit labeled with TRITC (SIGMA T5268), and 1:200 goat anti-chicken labeled with Dylight (JACKSON IMMUNO-RESEARCH, West Grove, USA, 103-495-1550)). They were rinsed in PBS five times and one time in 1 % PTA and pre-incubated for 60 min in Alexa Fluor 488-labeled phalloidin (0.33 M in 1 % PTA). Thereafter, specimens were rinsed in PBS (without NaN_3_) and mounted in Fluoromount-G with DAPI (SOUTHERN BIOTECHNOLOGY ASSOCIATES, Inc., Alabama, USA) or Vectashield with DAPI (VECTOR LABORATORIES, Burlingame, USA). The specificity of the antibodies was tested by examining specimens, where either the primary or secondary antibodies were omitted. Chicken anti acetylated α-tubulin staining did not produce satisfying results and is therefore not shown in this study (Sigma SAB3500023-100UG).

The mounted specimens were scanned using an Olympus Fluoview FV-1000 confocal laser scanning microscope (of K. Worsaae, University of Copenhagen, Denmark), with the acquired z-stacks of scans being either projected into 2D-images or analyzed three-dimensionally using IMARIS 7.1 (BITPLANE SCIENTIFIC SOFTWARE, Zürich, Switzerland). This software package was also used to conduct the measurements presented in the following text according to the conventions introduced by Hummon et al. [55], i.e. position in the body is given in units (U) as a relative measurements to total body length, measured from anterior to posterior. Schematic hand drawings and plate setup were done with Adobe Illustrator CS6 and image adjustments conducted in Adobe Photoshop CS6.

## Results

### Phylogeny

**Fig. 1:**
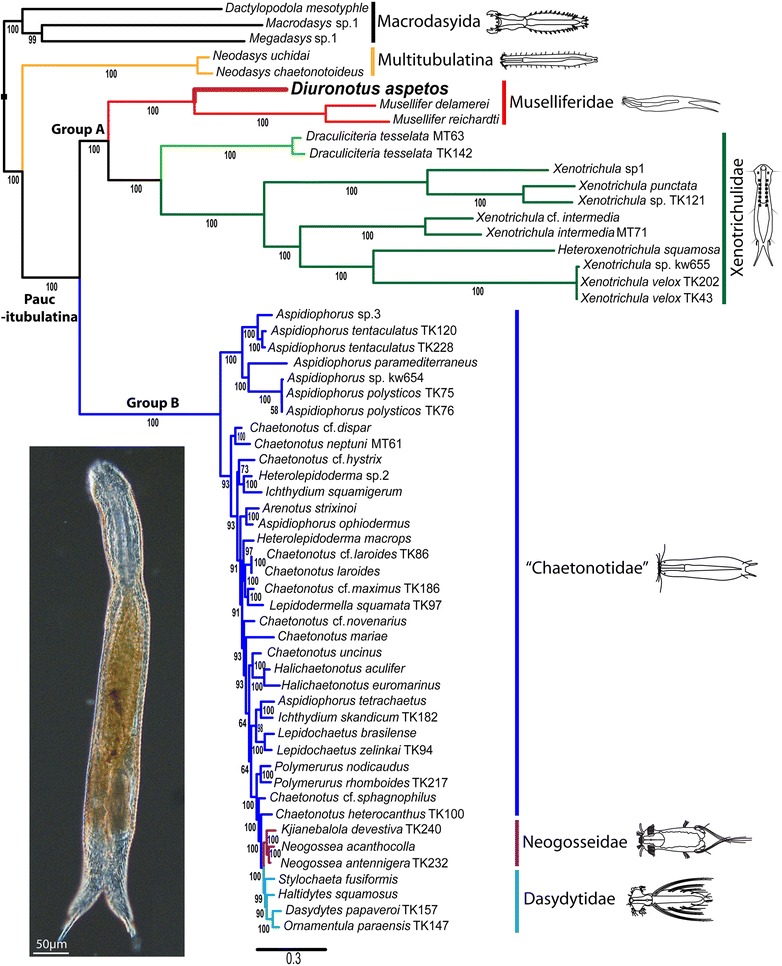
Phylogenetic position of *Diuronotus aspetos* inferred from Bayesian analysis of 18S, 28S, and COI. This tree is commented in the results section. The analysis includes 58 taxa representing all available genera of Chaetonotida for molecular data on NCBI, and three Macrodasyida as outgroups. Numbers at the nodes represent posterior probabilities in percentages. The picture on the lower left corner is a light micrograph of a live specimen of *Diuronotus aspetos*

The tree (Fig. 1) shows a fully supported sister group relationship (100 % posterior probability (PP)) between the monophyletic Muselliferidae (100 % PP) and Xenotrichulidae (100 % PP), herein collectively called “group A”. Within Muselliferidae the genus *Musellifer* (100 % PP) (represented only by *Musellifer delamarei* (Renaud-Mornant [56]) and *Musellifer reichardti* Kånneby et al. [26]) is found to be the sister group to *Diuronotus aspetos*. Group A is found next to “group B”, together constituting the Paucitubulatina (100 % PP), with group B showing a monophyletic Dasydytidae (100 % PP) and Neogosseidae (100 % PP) nested within “Chaetonotidae”, the latter hereby becoming paraphyletic. Supports are high in all nodes of the tree, except for some of the numerous inner nodes of group B.

### Musculature

**Fig. 2:**
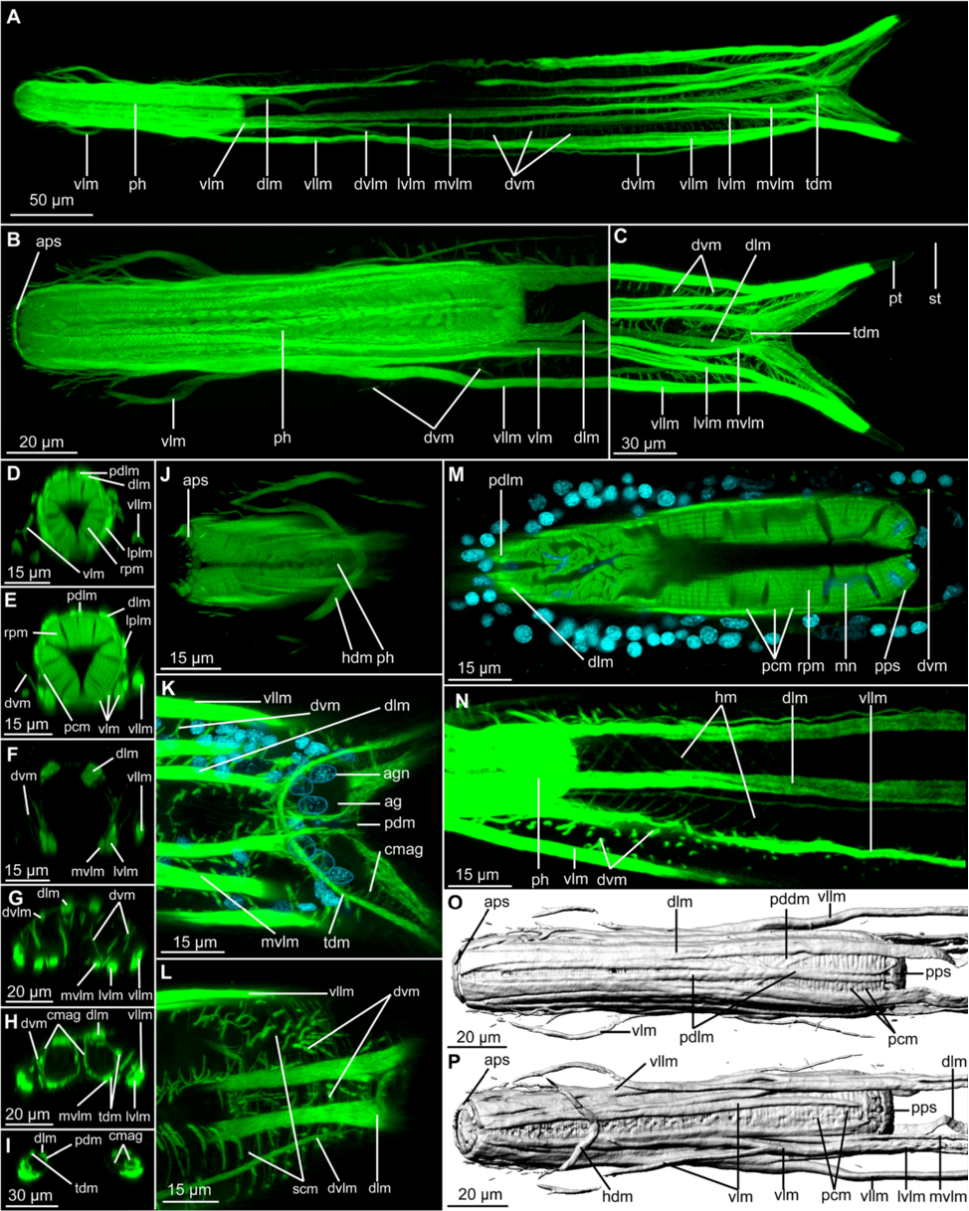
CLSM of phalloidin stained muscle of *Diuronotus aspetos*. Anterior of the specimen is pointing *left* for **a**, **b** and **j**–**p**, and dorsal is pointing at the *top* for **d**–**i**. **a**–**n** Muscles in *green*, nuclei in *cyan* **a** Ventral view of the maximum intensity projection (MIP) of the whole specimen. **b** Dorsal MIP of the pharynx. **c** Dorsal MIP of the posterior specimen. **d**–**i** CLSM virtual transverse section of various parts of the specimen: **d** head, **e** posterior part of the pharynx, **f** anterior of the trunk, **g** posterior of the trunk, **h** post-anal region of the trunk, **i** and furca before bifurcation of the tubes. **j** Dorsal MIP of a sub-stack showing details on the head musculature. **k** Dorsal MIP of a sub-stack showing details of the furca separation. **l** Ventral MIP of a sub-stack showing details of the semicircular musculature. **m** Single section showing details of the inner pharynx. **n** Dorsal MIP of a substack showing details of the helicoidal musculature. **o** and **p**, isosurface reconstruction of the pharynx. **o** Dorsal view, **p** ventral view. *ag* adhesive gland, *agn* adhesive gland nucleus, *aps* anterior pharyngeal sphincter, *cmag* circular muscle of the adhesive gland, *dlm* dorsal longitudinal muscle, *dvlm* dorsal projection of the ventral longitudinal muscle, *dvm* dorso-ventral muscle, *hdm* head diagonal muscle, *hm* helicoidal muscles, *lplm* lateral pharyngeal longitudinal muscle, *lvlm* Lateral extension of the ventral longitudinal muscle, *mn* myocyte nuclei, *mvlm* medial projection of the ventral longitudinal muscle, *pcm* pharyngeal circular muscle, *pddm* pharyngeal dorsal diagonal muscle, *pdm* posterior diagonal muscle, *pdlm* pharyngeal dorsal longitudinal muscle, *ph* pharynx, *pps* posterior pharyngeal sphincter, *pt* primary tube, *rpm* radial pharyngeal muscles, *scm* semi-circular muscle, *st* secondary tube, *tdm* tube diagonal muscle, *vlm* ventral longitudinal muscle, *vllm* ventro-lateral longitudinal muscle, *vlm* ventral longitudinal muscle

**Fig. 3:**
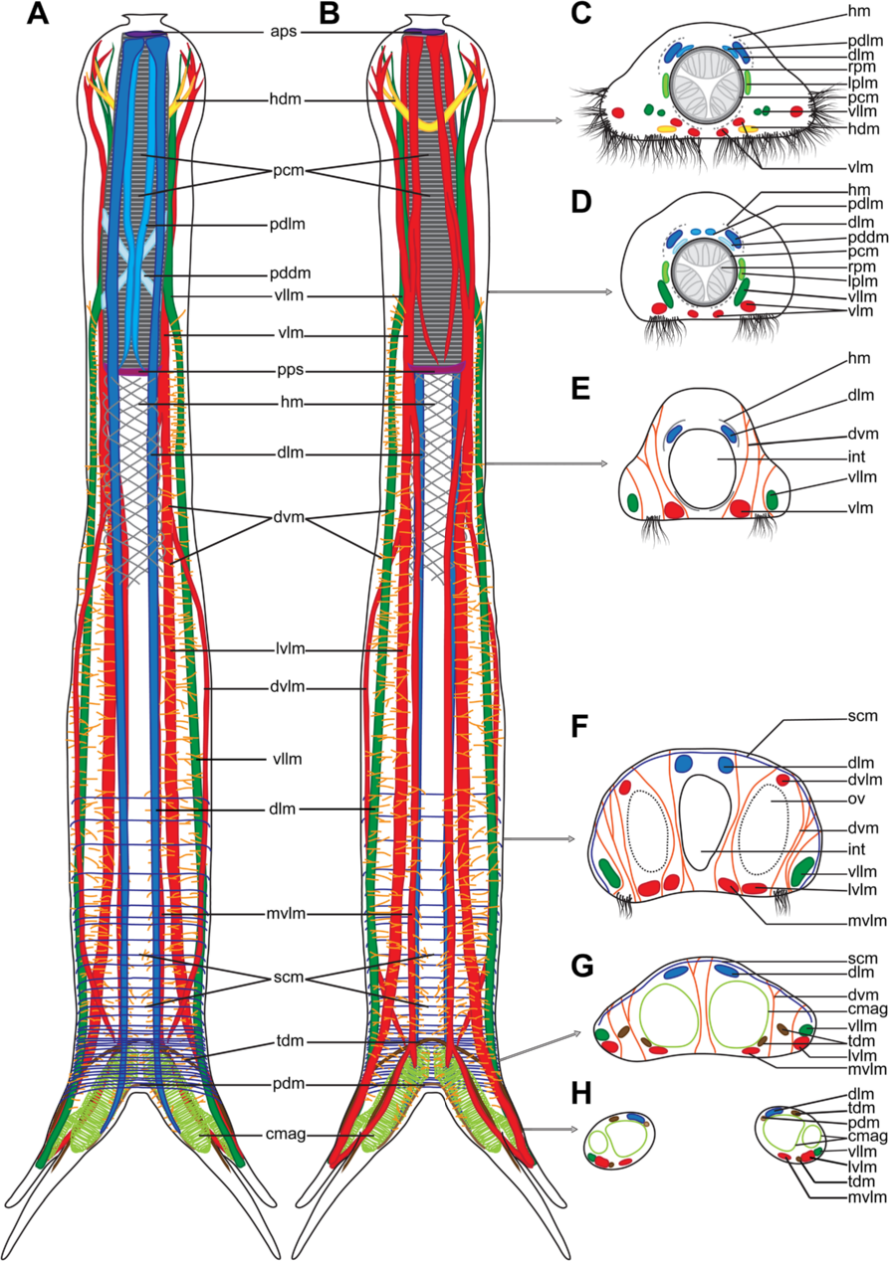
Schematic drawings of the musculature of *Diuronotus aspetos*. Anterior end is pointing at the *top* for **a** and **b**, dorsal side is pointing at the *top* for **c**–**h**. **a** Ventral view of the musculature, **b** dorsal view of the musculature, **c**–**h** cross section of the specimen, **c** in the head, **d** posterior part of the pharynx, **e** anterior of the trunk, **f** posterior of the trunk, **g** post-anal region of the trunk, **h** and in the furca before bifurcation of the tubes. Note that in **c** and **d**, the helicoidal pharyngeal musculature is represented in *dash lines* due to the uncertainty of its presence, and it is not drawn in (**a** and **b**). *aps* anterior pharyngeal sphincter, *cmag* circular muscle of the adhesive gland, *dlm* dorsal longitudinal muscle, *dvlm* dorsal projection of the ventral longitudinal muscle, *dvm* dorso-ventral muscle, *hdm* head diagonal muscle, *hm* helicoidal muscle, *int* intestine, *lplm* lateral pharyngeal longitudinal muscle, *lvlm* Lateral extension of the ventral longitudinal muscle, *mvlm* medial projection of the ventral longitudinal muscle, *ov* ovary, *pcm* pharyngeal circular muscle, *pdm* posterior diagonal muscle, *pddm* pharyngeal dorsal diagonal muscle, *pdlm* pharyngeal dorsal longitudinal muscle; *pps* posterior pharyngeal sphincter, *rpm* radial pharyngeal muscles, *scm* semi-circular muscle, *tdm* tube diagonal muscle, *vllm* ventro lateral longitudinal muscle, *vlm* ventral longitudinal muscle

The body wall musculature consists of several pairs of longitudinal muscles, numerous dorso-ventral muscles, a thin helicoidal musculature, semi-circular and complete circular muscles, as well as pharyngeal musculature. The pharyngeal musculature is especially dense and has an organization typical of chaetonotid gastrotrichs, as described below in more detail (Figs. 2 and 3).

#### Radial muscles

The pharynx, *sensu stricto*, is formed by three rows of dense radial pharyngeal muscles (rpm, Figs. 2d, e, m and 3c, d), and extend to U26 (units are calculated as length from anterior end, relative to total length, see material and methods). The radial muscles are cross-striated and each of them presents three to six Z-discs, which are less numerous anteriorly. The myoepithelial nuclei of the pharynx have a distinctive folded and elongated shape (mn, Fig. 2m). The pattern and repartition of these nuclei seems to be specific and corresponding nuclei could be found in the same position in different specimens.

#### Helicoidal muscles

Helicoidal muscles (hm, Figs. 2n and 3a, b, d, e) are very thin (0.5–1.2 μm) and limited to the anterior half of the specimen. It is difficult to confirm the presence of the helicoidal muscles closest to the pharynx due to the strong signal of other pharyngeal muscles (dashed lines in Fig. 3c-d). In a few locations along the midline of the pharynx very faint diagonal fibers were observed, suggesting that the helicoidal musculature is present along the entire pharyngeal region. Distinct helicoidal muscles are found extending from the midgut/pharynx junction at U26 until U42, enveloping the dorsal longitudinal muscle, but not the ventral or ventro-lateral longitudinal muscles.

#### Longitudinal musculature

Three longitudinal muscles span the entire body length: a pair of ventral longitudinal muscles (vlm, Figs. 2 and 3), a pair of ventro-lateral longitudinal muscles (vllm, Figs. 2 and 3), and a pair of dorsal longitudinal muscles (dlm, Figs. 2 and 3). The ventral longitudinal muscle bundle splits several times in a pattern described below for the different body regions.

##### Pharyngeal region

Several longitudinal muscles are present along the pharynx. Some are limited to the pharyngeal region, while others are the continuity of the body longitudinal muscles mentioned above. Two sets of muscles are strictly limited to the pharyngeal region: a pair of lateral and a pair of dorsal muscles. The lateral pharyngeal longitudinal muscle (lplm, Figs. 2d, e and 3c, d) extends adjacent to the pharynx along its entire length. The pharyngeal dorsal longitudinal muscles (pdlm, Figs. 2d, e, m, o and 3b-d) extend close to the pharyngeal midline along its total length, ventral to the dorsal longitudinal muscles. Moreover, several somatic and splanchnic longitudinal muscles supply the pharyngeal region.

The paired ventral longitudinal muscle (vlm, Figs. 2 and 3) originating in the head splits along the pharynx into a complex pattern (see Fig. 3b). One of its branches extends more laterally and splits into several sub-branches, supplying the lateral sides of the head.

The paired ventro-lateral longitudinal muscle (vllm, Figs. 2 and 3) lines the pharynx until reaching the head, where it bifurcates at U7, one branch extending ventro-laterally and the other dorso-laterally. Each of them subsequently splits into several minor branches, supplying the lateral sides of the head. These muscles together with the antero-lateral branch of the ventral longitudinal muscle (vlm), and the head diagonal muscle (hdm, see below) all supply the antero-lateral part of the head, and in addition to anchoring the longitudinal muscles for overall body contraction, they may function separately in contraction of the head (Figs. 2j and 3a, c).

The paired dorsal longitudinal muscle (pdlm) spans the anterior-most extremity of the pharynx.

##### Trunk region

Three main longitudinal muscles, i.e. ventral, lateral and dorsal, are supplying the trunk. The paths of the lateral and dorsal longitudinal muscles are relatively straight throughout the body. However, just posterior to the pharynx, the dorsal longitudinal muscle lines the intestine (Figs. 2f and 3c, d, e), while it runs closer to the dorsal body wall more posteriorly (Figs. 2g, h and 3f, g). The ventral longitudinal muscle splits into three muscle bundles at the anterior trunk. Two of these branches run in parallel mid-ventrally along the trunk (lvlm, mvlm, Figs. 2a, c, f-h, p and 3a, b, f-h), whereas the third branch extends dorso-laterally and supplies the dorso-lateral sides of the body until meeting the median-most branch at U86 (dvlm, Figs. 2a, g, l and 3a, b, f).

##### Posterior region

The median branch of the longitudinal ventral muscle splits posteriorly into two bundles at U86. One very short (8 μm) portion medially supplies the posterior part of the adhesive gland of the posterior tube, while another longer branch extends into the primary tube, supplying it for approximately two-thirds of its length to U97 (Fig. 3a). Additionally, the lateral branches of the ventral and lateral longitudinal muscles also extend along the primary tube. The dorsal longitudinal muscle supplies the anterior third of the secondary tube.

#### Diagonal muscles

The head diagonal muscle (hdm, Figs. 2j, p and 3a, b, c) forms a V-shape with two medially joined branches. The median part of the muscle is situated in the midline of the body in the posterior region of the head while the two extremities extend to the antero-lateral region of the head.

At the dorso-posterior pharynx, a pair of pharyngeal dorsal diagonal muscles decussate (pddm, Figs. 2p and 3b, d), ventral to the pharyngeal dorsal longitudinal muscle and the dorsal longitudinal muscle. Although their orientation is similar to that of the helicoidal muscles, their exclusively dorsal extension and their greater width differ significantly from those of the helicoidal muscles.

Three diagonal muscles are found in the furca, extending from one side to the contralateral one. The two anterior muscles (tmd, Figs. 2a, b, h, i, k and 3a, b, g, h) extend from the midline at U86, halfway to the primary and the secondary tube, respectively. The posterior diagonal muscle (pdm, Figs. 2i, k, and 3a, b, h) extends from U89 laterally into the first third of the secondary tube.

#### Circular muscles

Pharyngeal circular muscles (pcm 2E,M,O,P and 3A,B,C,D) are present around the pharynx. These muscles are numerous (ca. 110 in one specimen), positioned proximal to each other, and 1–1.50 μm thick with increasing diameter towards the posterior region.

Two sphincters are present at each extremity of the pharynx: one anterior pharyngeal sphincter (aps, Figs. 2b, o, p and 3a, b) located just posterior to the mouth, and one posterior pharyngeal sphincter (pps, Figs. 2m, o, p and 3a, b) marking the transition between the intestine and the pharynx. The anterior sphincter is smaller, being 1 μm thick and has a diameter of 17 μm, while the posterior sphincter is more prominent with 8 μm thickness and a diameter of 23 μm.

Supplementary circular muscles of the adhesive glands (cmag, Figs. 2h, i, k and 3a, b, g, h) are present in the tubes, forming a muscular layer around the large adhesive glands (ag, Fig. 2k). They thereby supply two cavities, one for each tube. The muscular layer surrounding the primary tube is smaller than the one surrounding the secondary tube, and both structures are connected. This suggests that both tubes are supplied by a single set of glands controlled by muscles. Three adjacent nuclei are found within the layer of circular muscles at the base of the gland (agn, Fig. 2k), near the anus, around U88.

#### Semi-circular muscles

Ventrally opened semicircular muscles (scm, Figs. 2l and 3a, b, f, g) are present in the posterior part of the specimen, but do not extend into the tubes. They originate ventrally, from each side of the body, and extend to the dorsal side. From there, they project to the contralateral side, external to the longitudinal musculature. They are more numerous in the posterior region, anterior to the furca, where they are separated by 2–5 μm. Semicircular muscles are less numerous and spaced further apart (5–8 μm) in the anterior region of the ovary. They appear to supply only the ovary region; their contraction probably reduces the body diameter and may be involved in the movement/release of eggs.

#### Dorso-ventral muscles

Numerous thin muscles (1–2 μm in diameter) traverse the entire trunk dorso-ventrally (dvm, Figs. 2a–c, e-h, k, l, n and 3a, b, e-g). These dorso-ventral muscles are spaced approximately 5 μm apart in the region between U18 and U95. In this region, two pairs are found laterally in transverse sections of the pharyngeal region (one external and one more internal pair, dvm, Figs. 2d, e and 3a, b). This number increases more posteriorly in the trunk, where up to five pairs of dorso-ventral muscles can be detected (dvm, Figs. 2g, k, l, and 3a, b, f). The dorso-ventral muscles extend dorso-ventrally between the different longitudinal muscles and the ciliary bands in various combinations. However, they are never found external to the ventro-lateral longitudinal muscles or between the pair of dorsal longitudinal muscles.

### Nervous system

**Fig. 4:**
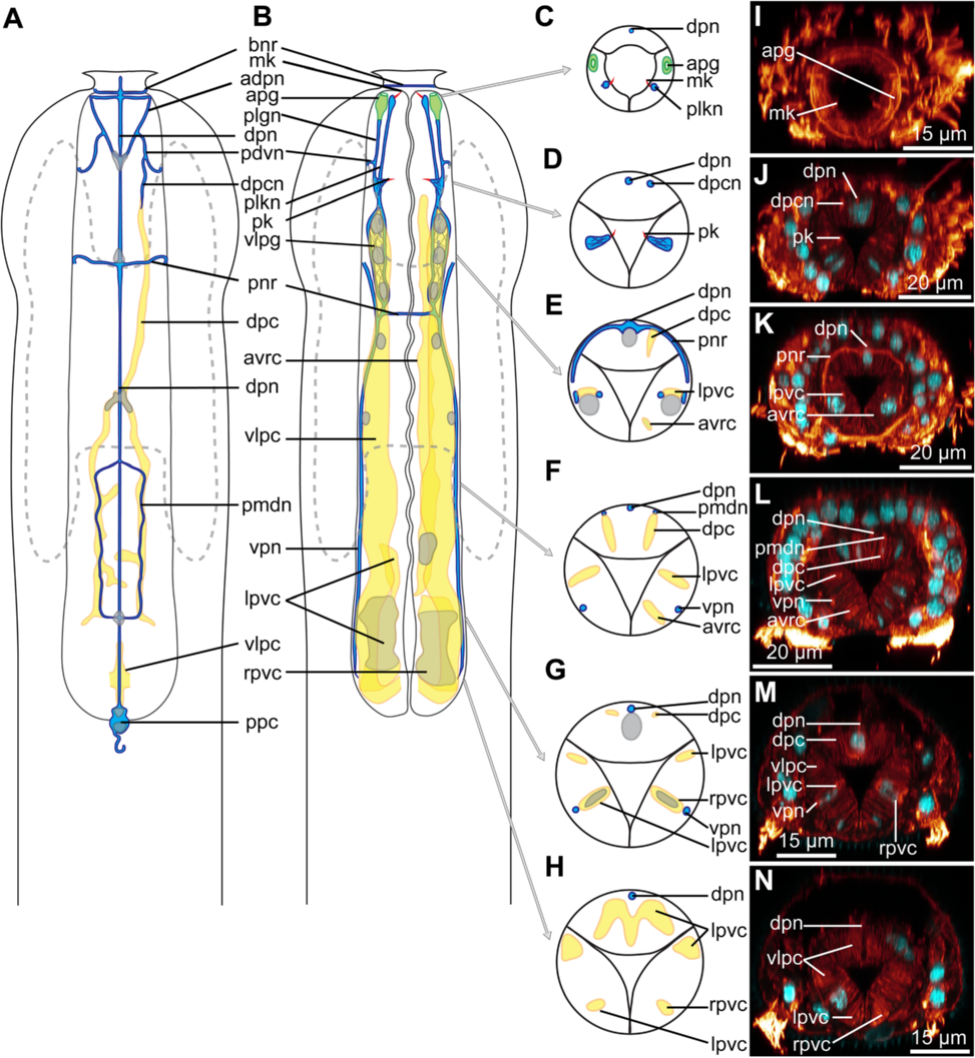
Pharyngeal nervous system and canal system of *Diuronotus aspetos*. **a**, **b** Anterior is pointing at the *top*; **c**–**n** dorsal is pointing at the *top*. **a**–**h** Schematic drawings with nerves in *blue* and pharyngeal system in *yellow*, nuclei in *grey*, glands in *green* and cilia in *red*. **a** Dorsal section of the pharynx. **b** Ventral section of the pharynx. **c**–**h** Successive transverse sections of the pharynx from anterior to posterior. **i**–**n** CLSM virtual transverse sections at the same levels as (**c**–**h**). Acetylated α-tubulin-LIR in *glow* and DAPI in *cyan*. *adpn*, anterior diagonal pharyngeal nerve, *apg* anterior pharyngeal gland, *avrc* anterior ventro-median right pharyngeal canal, *bnr* buccal nerve ring, *dpc* dorsal pharyngeal canal, *dpcn* dorso-anterior pharyngeal canal nerve, *dpn* dorsal pharyngeal nerve, *lpvc* left posterior ventro-median canal, *mk* mouth kinocilium, *pdvn* pharyngeal dorso-ventral nerve, *pk* posterior pharyngeal kinocilium, *plgn* pharyngeal longitudinal gland nerve, *plkn* pharyngeal longitudinal kinocilium nerve, *pmdn* paramedian dorsal pharyngeal nerves, *pnr* pharyngeal nerve ring, *ppc* posterior pharyngeal cluster, *rpvc* right posterior ventro-median canal, *vlpc* ventro-lateral pharyngeal canal, *vlpg* ventro-lateral pharyngeal ganglion, *vpn* ventral pharyngeal nerve

**Fig. 5:**
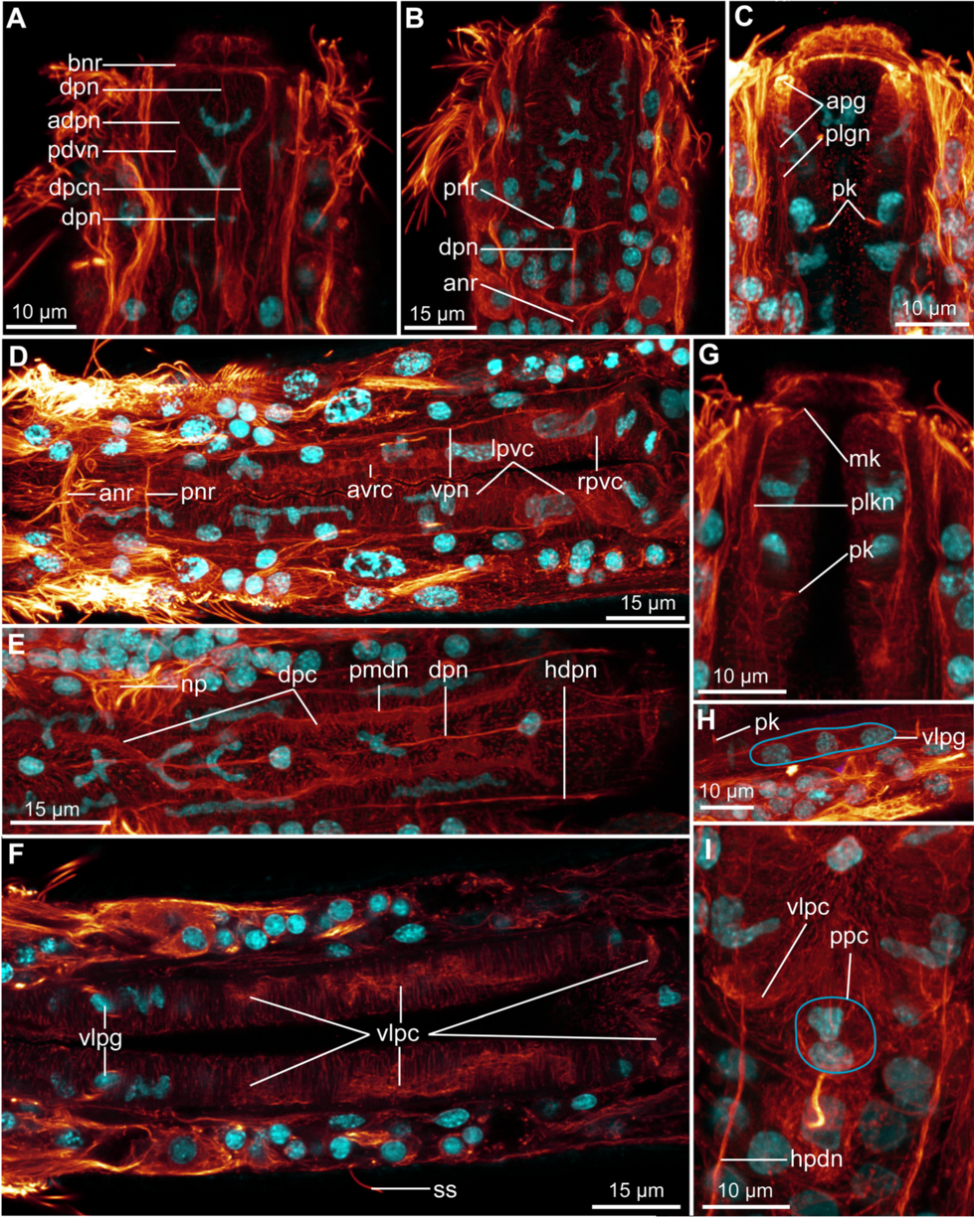
CLSM of the pharyngeal nervous system and canal system of *Diuronotus aspetos*. **a**–**c**, **g**, **i** anterior is pointing at the *top*. **d**–**f**, **h** anterior pointing *left*. CLSM maximum intensity projection of sub-stacks. Acetylated α-tubulin-LIR in *glow*, DAPI in *cyan*. **a** Dorso-anterior section of the pharynx. **b** Dorso-anterior section of the pharynx, more ventral than **b**). **c** Ventro-anterior section of the pharynx. **d** Ventro-posterior section of the pharynx. **e** Dorso-posterior section of the pharynx. **f** Medio-posterior portion of the pharynx. **g** Medio-anterior section of the pharynx. **h** Details of the ventro-lateral pharyngeal ganglion. **i** Details of the posterior pharyngeal ganglion. *adpn* anterior diagonal pharyngeal nerve, *anr* anterior nerve ring, *apg* anterior pharyngeal gland, *avrc* anterior ventro-median right pharyngeal canal, *bnr* buccal nerve ring, *dpc* dorsal pharyngeal canal, *dpcn* dorso-anterior pharyngeal canal nerve, *dpn* dorsal pharyngeal nerve, *hdpn* head dorso-posterior nerve, *lpvc* left posterior ventro-median canal, *mk* mouth kinocilium, *np* neuropile, *pdvn* pharyngeal dorso-ventral nerve, *pk* posterior pharyngeal kinocilium, *plgn* pharyngeal longitudinal gland nerve, *plkn* pharyngeal longitudinal kinocilium nerve, *pmdn* paramedian dorsal pharyngeal nerves, *pnr* pharyngeal nerve ring, *ppc* posterior pharyngeal cluster, *rpvc* right posterior ventro-median canal, *ss* sensoria, *vlpg* ventro-lateral pharyngeal ganglion, *vlpc* ventro-lateral pharyngeal canal, *vpn* ventral pharyngeal nerve

**Fig. 6:**
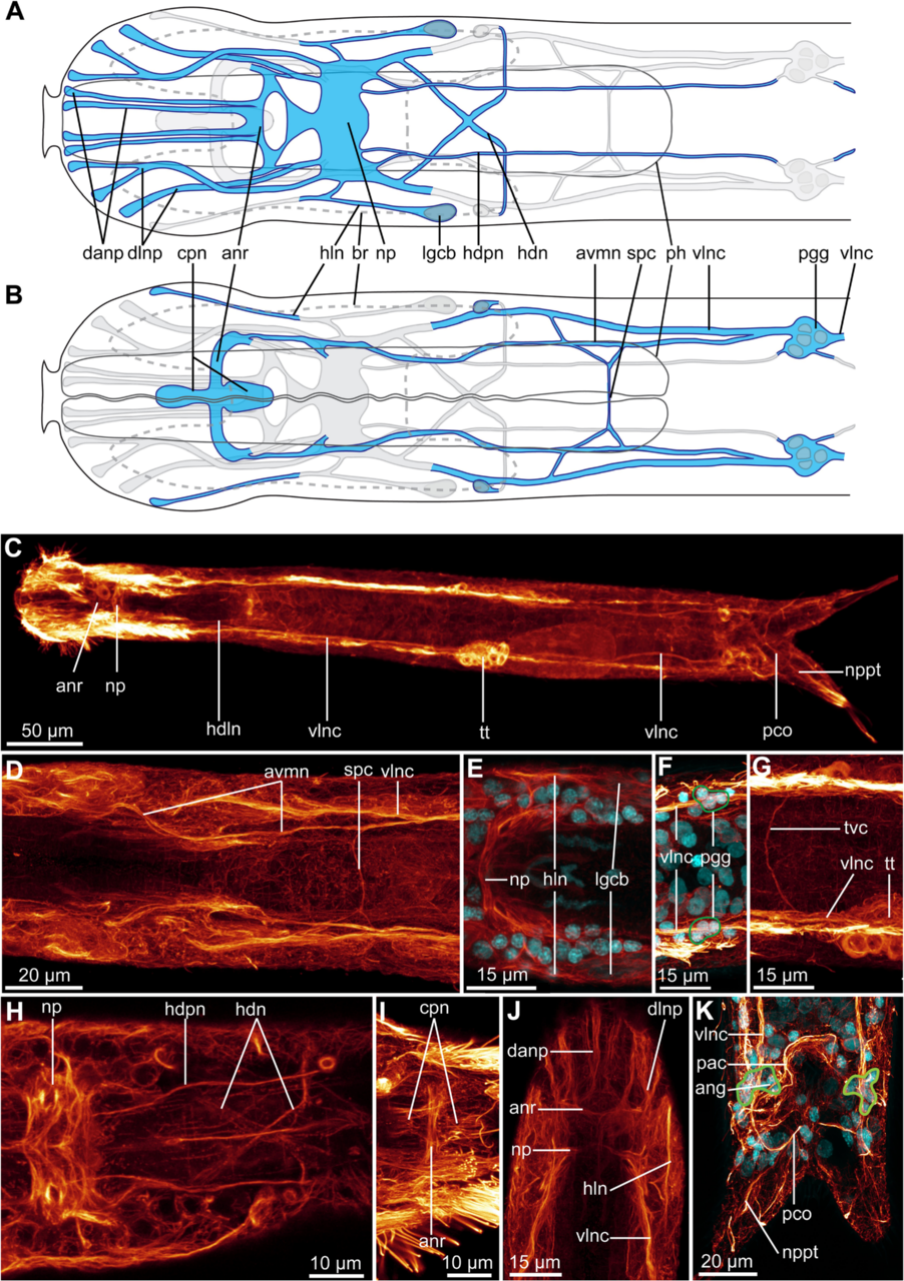
Drawing and CLSM of the acetylated α-tubulin-LIR nervous system of *Diuronotus aspetos*. Anterior pointing *left* for **a**–**i**, and pointing at the *top* for **j** and **k**. **a**, **b** Schematic drawings of the α-tubulin-LIR of the anterior part of the specimen: nerves in *blue*, nuclei in *grey*, and opposite ventral or dorsal nervous system in *light grey* **a** dorsal **b** ventral. **c** CLSM ventral view of the maximum intensity projection (MIP) of the entire specimen. **d**–**k** CLSM MIP sub-stacks of various parts of the specimen. Acetylated α-tubulin-LIR in *glow*, and DAPI in *cyan* in all CLSM pictures. **d** Ventro-anterior nervous system. **e** Neuropil side **f** Ventral, post pharyngeal ganglion. **g** Ventral, trunk commissure. **h** Dorso-posterior part of the head **i** ventro-anterior part of the head **j** Dorso-anterior part of the head. **k** Ventro posterior terminal part of the specimen. *ang* anal ganglion, *anr* anterior nerve ring, *avmn* anterior ventro-median nerve, *br* brain, *cpn* ciliated patch nerves, *danp* dorso-median anterior nerve projection, *dlnp* dorso-lateral anterior nerve projections, *hdpn* head dorso-posterior nerve, *hdn* head diagonal nerve, *hln* head lateral nerve, *lgcb* lateral gland cell of the brain, *np* neuropile, *nppt* nerve projection of the primary tube, *pac* pre-anal commissure, *pco* posterior commissure, *pgg* post-pharyngeal ganglion, *ph* pharynx, *spc* sub-pharyngeal commissure, *tt*, testis, *tvc* trunk ventral commissure, *vlnc* ventro-lateral nerve cord

**Fig. 7:**
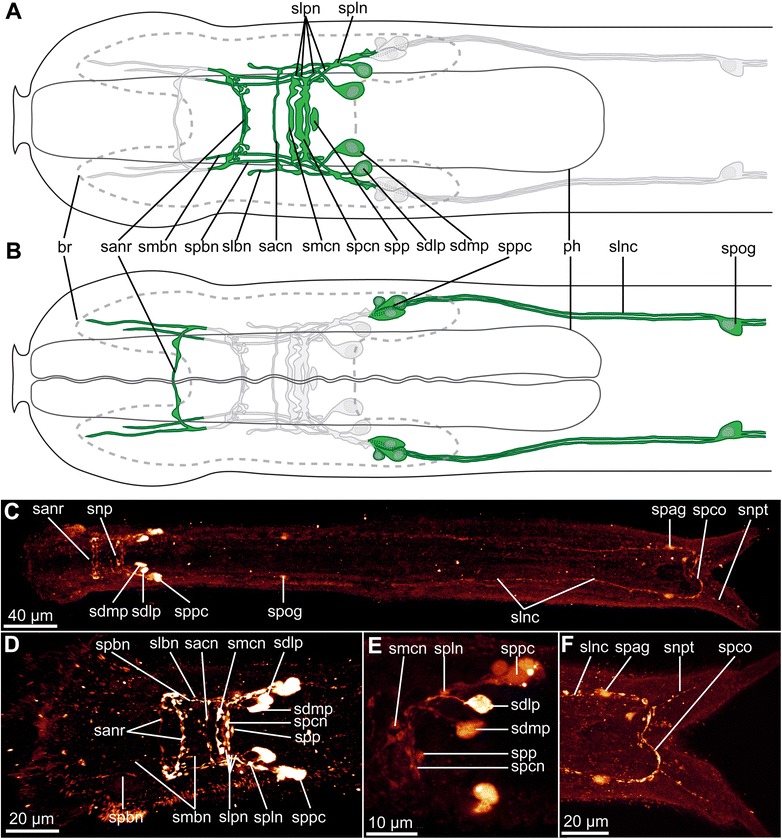
serotonin-LIR nervous system of *Diuronotus aspetos*. The anterior is pointing *left* for all figures. **a**, **b** Schematic drawings of the serotonin-LIR of the anterior part of the specimen: nerves and perikarya in *green*, nuclei in *grey*, and opposite ventral or dorsal nervous system in *light grey*. **a** Dorsal view, **b** ventral view. **c**–**f** CLSM images with serotonin-LIR in *glow*. **c** CLSM maximum intensity projection (MIP) of the entire specimen. **d** Dorsal MIP of the brain **e** CLSM sub-stack MIP showing details of the brain perikarya **f** CLSM sub-stack MIP of the ventro-posterior terminal part of the specimen. *br* brain, *ph* pharynx, *sacn* serotonin-LI-reactive anterior commissure of the neuropil, *sanr* serotonin-LI-reactive anterior nerve ring, *sdlp* serotonin-LI-reactive dorso-lateral perikaryon, *sdmp* serotonin-LI-reactive dorso-median perikaryon, *slbn* serotonin-LI-reactive lateral brain nerve, *slnc* serotonin-LI-reactive ventro-lateral nerve cord, *slpn* serotonin-LI-reactive lateral nerves of the posterior commissure of the neuropil, *spln* serotonin-LI-reactive postero-lateral nerve node, *smbn* serotonin-LI-reactive median-most brain nerve, *smcn* serotonin-LI-reactive median commissure of the neuropil, *snp* serotonin-LI-reactive neuropil, *snpt* serotonin-LI-reactive nerve projection of the primary tube, *spag* serotonin-LI-reactive perikarya of the anal ganglion, *spbn* serotonin-LI-reactive paramedian brain nerve, *spcn* serotonin-LI-reactive posterior commissure of the neuropil, *spco* serotonin-LI-reactive posterior commissure, *spog* serotonin-LI-reactive perikarya of the post-pharyngeal ganglion, *spp* serotonin-LI-reactive neuropil patch, *sppg* serotonin-LI-reactive para-pharyngeal cluster

**Fig. 8:**
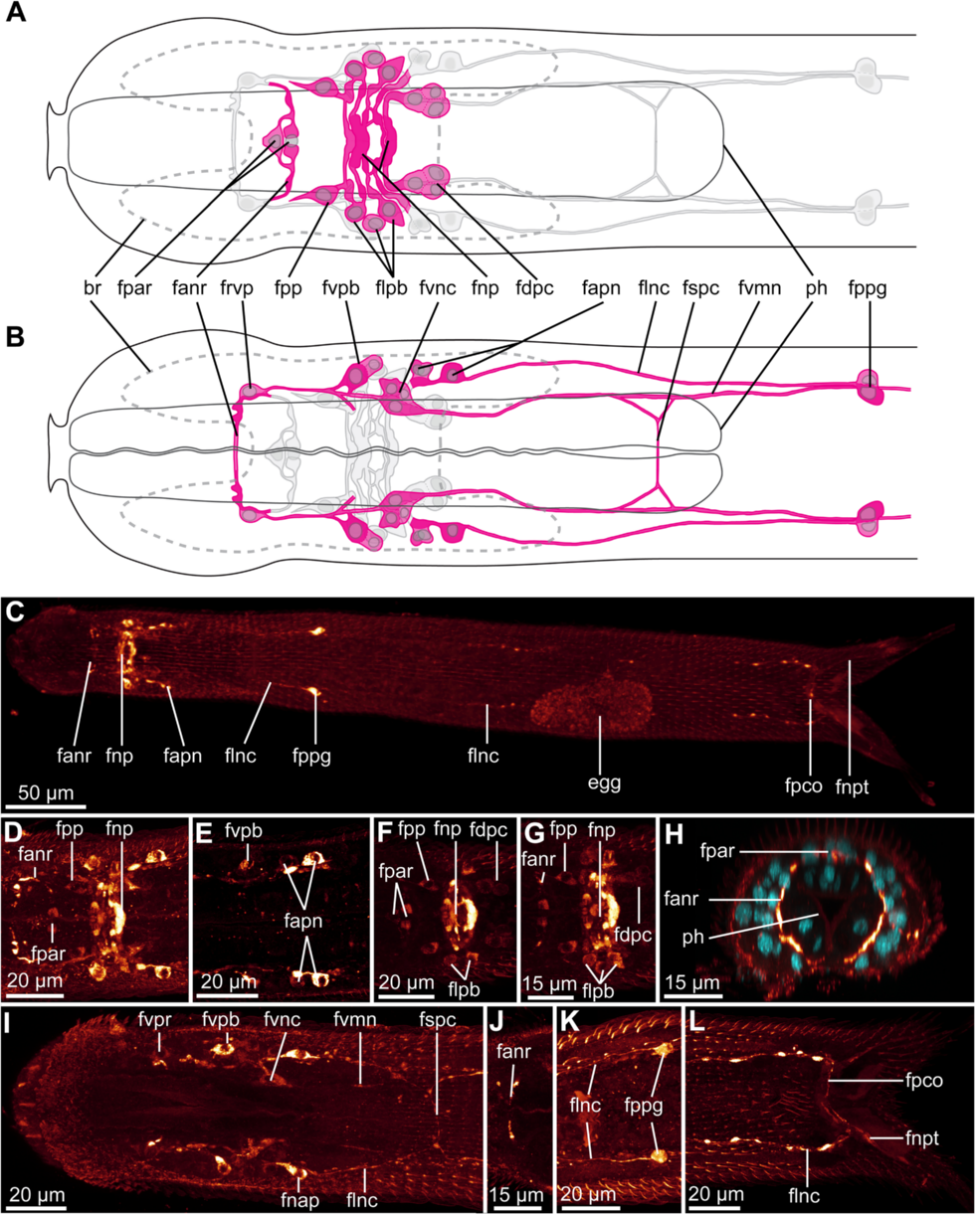
FMRF-amide-LIR nervous system of *Diuronotus aspetos*. Anterior is pointing *left* for **a**–**g** and **i**–**l** and dorsal pointing at the *top* for **h**. **a**, **b** Schematic drawings of the FMRF-amide-LIR of the anterior part of the specimen: nerves in *magenta*, nuclei in *grey*, and opposite ventral or dorsal nervous system in *light grey*. **a** Dorsal view, **b** ventral view. **c** CLSM dorsal view of the maximum intensity projection (MIP) of the entire specimen. **d**–**l** (Except **h**) CLSM sub-stack MIP of various parts of the specimen. FMRF-amide-LIR in *glow*, and DAPI in *cyan* in all CLSM pictures. **d** Dorsal view of the whole neuropil. **e** Ventral part of the brain. **f** and **g** different levels of the dorsal part of the neuropil. **h** CLSM virtual transverse section of the anterior nerve ring. **i** Ventro-anterior part of the head. **j** Ventral commissure of the anterior nerve ring. **k** Ventral post-pharyngeal ganglia. **l** ventro-posterior terminal part of the specimen. Anterior of the specimen on the left for **a**-**g** and **i**-**l** and dorsal on top for **h**. *br* brain, *egg* egg, *fanr* FMRF-amide-LI-reactive anterior nerve ring, *fapn* FMRF-amide-LI-reactive anterior perikarya of the ventro-lateral nerve cord, *fdpc* FMRF-amide-LI-reactive dorso-posterior cluster of the brain, *flnc* FMRF-amide-LI-reactive ventro-lateral nerve cord, *flpb* FMRF-amide-LI-reactive lateral perikarya of the brain, *fnp* FMRF-amide-LI-reactive neuropil, *fnpt* FMRF-amide-LI-reactive nerve projection of the primary tube, *fpar* FMRF-amide-LI-reactive dorso-median perikarya of the anterior nerve ring, *fpco* FMRF-amide-LI-reactive posterior commissure, *fpp* FMRF-amide-LI-reactive perikarya of the dorso-lateral anterior nerve projections, *fppg* FMRF-amide-LI-reactive post-pharyngeal ganglion, *fspc* FMRF-amide-LI-reactive sub-pharyngeal commissure, *fvmn* FMRF-amide-LI-reactive anterior ventro-median nerve, *fvnc* FMRF-amide-LI-reactive anterior ventro-median nerve cluster, *fvpb* FMRF-amide-LI-reactive ventro-lateral perikarya of the brain, *fvpr* FMRF-amide-LI-reactive ventral perikarya of the anterior nerve ring, *ph* pharynx

**Fig. 9:**
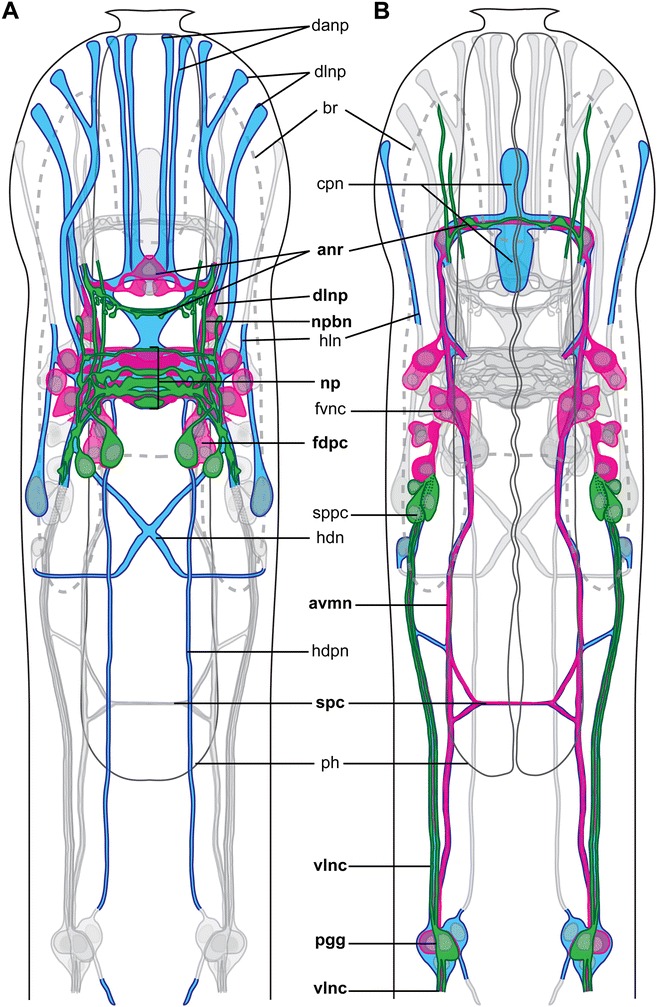
Schematic drawing of acetylated α-tubulin-LIR, FMRF-amide-LIR and serotonin-LIR nervous system of *Diuronotus aspetos,* showing the correspondences between the different nervous systems. Anterior pointing at the *top*. Acetylated α-tubulin-LIR nervous system in *blue*, FMRF-amide-LIR nervous in *magenta*, and serotonin-LIR nervous system in *green*. Cell nuclei in *grey* and opposite nervous system in *light grey*. Legends in *bold* indicate structures showing-LIR for at least two molecules tested. **a** Dorsal, and **b** ventral. *anr* anterior nerve ring, *avmn* anterior ventro median nerve cord, *br* brain, *cpn* ciliated patch nerves, *danp* dorso-median anterior nerve projection, *dlnp* dorso-lateral anterior nerve projections, *fdpc* FMRF-amide-LI-reactive postero-lateral brain cluster, *fvnc* FMRF-amide-LI-reactive ventro-median nerve cluster, *hdn* head diagonal nerve, *hln* head lateral nerve, *hdpn* head dorso-posterior nerve, *hpdn* head diagonal nerve, *np* neuropile, *pgg* post-pharyngeal ganglion, *ph*, pharynx, *spc* sub-pharyngeal commissure, *sppc* serotonin-LI-reactive para-pharyngeal cluster, *vlnc* ventro-lateral nerve cord

The nervous system of *Diuronotus aspetos* is described from acetylated α-tubulin-like immunoreactivity (LIR, Figs. 4, 5 and 6), serotonin-LIR (Fig. 7) and FMRF-amide-LIR (Fig. 8) (all different LIR of the head region are summarized in Fig. 9). Similar to previously investigated Gastrotricha, the nervous system consists of paired nerve cords, which originate from a bilobed dorsal brain, and extend posteriorly. In the following section, previously described and undescribed structures are detailed, such as: i) multiple pairs of longitudinal nerve projections in the head (danp, dlpn, hln, Figs. 6a, b, e, j and 9a, b); ii) paired anterior ventro-median nerves (avmn, Fig. 6a, d and 9b); iii) dorsal nerves posterior to the brain (hdpn, Figs. 6a, h and 9a); iv) paired ventro-lateral nerve cords (vlnc, Figs. 6b-d, f, g, k and 9b); v) paired posterior nerves, projecting into the adhesive tubes (nppt, Fig. 6k), vi) bilobed, dorsal brain with three commissures (main neuropil and anterior ventral and dorsal commissure, together forming a nerve ring encircling the pharynx) (np and anr, Figs. 6a-c, e, h-j and 9a, b); vii) two pairs of ganglia along the nerve cord: one anterior and one terminal (pgg and ang, Figs. 6b, f, k and 9b); viii) four ventral trunk commissures (spc, tvc, pac and pco, Figs. 6b, d, g, k and 9b); and ix) a pharyngeal nervous system, consisting of three longitudinal nerves (one per pharyngeal row of radial muscles) and supplementary minor nerves (Figs. 4 and 5). Additionally, the serotonin-LIR and FMRF-amide-LIR gave very detailed results, allowing us to collect precise data on the number, position and connection of perikarya (Figs. 7, 8 and 9).

#### Acetylated α-tubulin-like immunoreactivity (acetylated α-tubulin-LIR)

Acetylated α-tubulin-LIR provides information on most neurites, cilia as well as other portions of cytoskeletons of the cells. However, not all minor neurites of the nervous system are traced and the description focuses on the central nervous system and sensory structures.

##### Stomatogastric nervous system

The stomatogastric nervous system, confined to the pharynx, consists mainly of three main longitudinal nerves: a dorsal (dpn, Figs. 4a, d–h, j–n and 5a, b, e) and two ventro-lateral nerves (vpn, Figs. 4b, f–h, l–n and 5e), extending basally along the midline of each row of radial muscles (Fig. 4). The nerves are closely related to three structures: kinocilia, anterior pharyngeal glands and a pharyngeal canal system. The dorsal pharyngeal nerve (dpn, Figs. 4a, d–h, k–o and 5a, b, e) originates at the mouth, where it supplies a buccal nerve ring (bnr, Figs. 4a, b and 5a), encircling the mouth (probably innervating the anterior sphincter (aps, Figs. 2b, o, p and 3a, b) opening and closing the mouth). At U4, two anterior diagonal pharyngeal nerves (adpn Figs. 4a and 5a) originate from the dorsal nerve, extend antero-laterally to the anterior edges of the pharynx and medially join back the dorsal nerve. At U3, one pharyngeal dorso-ventral nerve (pdvn, Figs. 4a, b and 5a) originates from each of the anterior diagonal nerve, and supply ventrally a pharyngeal gland longitudinal nerve (plgn, Figs. 4b and 5c) innervating an anterior pharyngeal gland (apg, Figs. 4a, c, i and 5c) (which opens into the mouth). On the right side of the specimen, a dorso-anterior pharyngeal canal nerve (dpcn, Figs. 4a, d, j and 5a) extends posteriorly from the anterior diagonal nerve, possibly supplying the asymmetric dorsal pharyngeal canal (dpc, Figs. 4a, f, g, l, m and 5e). At U9 a pharyngeal nerve ring (pnr, Figs. 4a, b, e, k and 5b, d) supplying the ventral and dorsal nerves is present. A pair of paramedian dorsal pharyngeal nerves (dpnp, Figs. 4a, f, l and 5e) originates from the dorsal nerve at U19 and extends in a parallel fashion on each side, to fuse again with the dorsal nerve at U26 (Figs. 4a, g, m and 5e). The dorsal nerve extends more posteriorly where it innervates a two-celled pharyngeal posterior cluster (ppc, Figs. 4a and 5i) at the posterior margin of the pharynx. Two nerves extend the terminal part of the ventro-lateral pharyngeal sections: the lateral gland longitudinal nerve (plgn, Figs. 4a and 5c), and a median kinocilium longitudinal nerve (plkn, Figs. 4b, c and 5g), with the latter supplying a mouth and a pharyngeal kinocilia, respectively, at U1 and U6 (mk and pk, Figs. 4b-d, j, i and 5c, g). The gland and kinocilium nerves originate at U7 from an elongated ventro-lateral pharyngeal ganglion (vlpg, Figs. 4 and 5h, f), consisting of three nuclei and extending from U7 to U12 (probably integrating the signal collected by the two kinocilia and responsible for the putative terminal gland secrete release). Moreover, the ganglion seems to be related to the ventro-lateral pharyngeal canal (vlpc, Figs. 4a, b, e-g, k-n and 5f, i) described below. The ventral pharyngeal nerves (vpn, Figs. 4b, f, g, l, m and 5d) (supplied by perikarya at U15 and U19) extend from the ganglion, until U28.

Due to the unknown nature of the canal system and its main acetylated α-tubulin-LIR (as well as a weak FMRF-amide-LIR), it is described in this nervous system section. It consists of radially flattened cavities, sometimes asymmetrical, extending longitudinally within the pharynx (Figs. 4a, b, e, f, k, l and 5d, e). Six pharyngeal canals extend the pharynx: i) the unpaired right ventro-anterior pharyngeal canal (avrc, Figs. 4b, e, f, k, l and 5d) extending from U6 to U26; ii–iii) The paired ventro-lateral pharyngeal canals (vlpc, Figs. 4a, b, e-h, k-n and 5f, i), extending from U7 (at the level of the pharyngeal ganglia (vlpg, Figs. 4a and 5h, f)) to U30, and merging dorso-posteriorly; iv-v) the paired ventro-posterior pharyngeal canals extending from U24 for the left one (lpvc, Figs. 4b, g, h, m, n and 5d) to U28 and from U26 for the right one (rpvc, Figs. 4b, g, h, m, n and 5d) to U28; vi) the dorsal pharyngeal canal (dpc, Figs. 4a, f, g, l, m and 5e) extending along the right side from U6 to U15, then reaching the midline and bifurcating in two symmetrical branches, following the paramedian dorsal nerves of the pharynx between U19 and U27. Few nuclei are embedded in the pharyngeal canal system (Fig. 4a, b, g).

##### Central nervous system

**Fig. 10:**
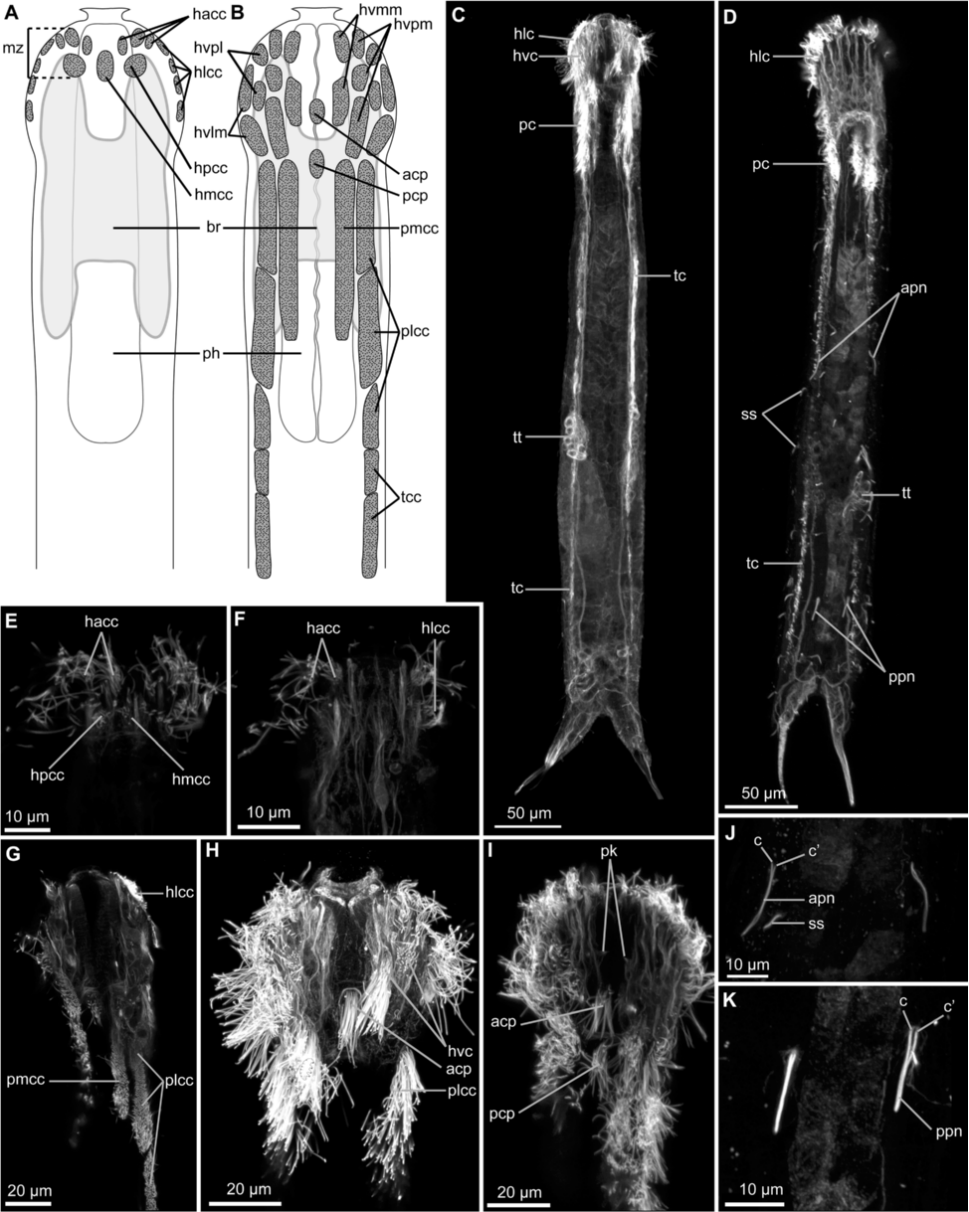
Ciliation of *Diuronotus aspetos*. Anterior pointing at the *top* for all figures. **a** and **b** drawings of the locomotory ciliation: **a** dorsal view, **b** ventral view. **c**–**k** CLSM maximum intensity projection (MIP) sub-stacks of the acetylated α-tubulin-LIR. **c** Ventral view of the whole specimen showing the organization of the locomotor ciliation. **d** Dorsal view of the whole specimen showing parts of the locomotor ciliation and the position of the protonephridia. **e** And **f**, dorsal head ciliation. **e** Is more dorsal than **f**. **g**–**i** Ventral head and pharyngeal ciliation: **g** is more dorsal than **h** which is more dorsal than **i**. **j** and **k** details of, respectively, the anterior and the posterior pairs of protonephridia. *acp* anterior ciliated patch, *apn* anterior proto-nephridia, *br* brain, *c, c’* cilia of the proto-nephridia, *hacc* head dorso-anterior ciliated cells, *hlc* head lateral ciliation, *hlcc* head lateral ciliated cells, *hmcc* head dorso-median ciliated cell, *hpcc* head postero-lateral dorsal ciliated cell, *hvc* head ventral ciliation, *hvlm* head ventral lateral-most row of ciliated cells, *hvmm* head ventral median-most row of ciliated cells, *hvpl* head ventral para-lateral row of ciliated cells, *hvpm* head ventral paramedian row of ciliated cells, *mz* muzzle, *pc* pharyngeal ciliation, *pcp* posterior ciliated patch, *ph* pharynx, *pk* posterior pharyngeal kinocilium, *plcc* pharyngeal lateral ciliated cells, *pmcc* pharyngeal median ciliated cell, *ppn* posterior proto-nephridia, *ss* sensoria, *tc* trunk ciliation, *tcc* trunk ciliated cells, *tt* testis

The neuropil (np, Figs. 6a, c, e, h, j and 9a) is 14 μm thick and its center is positioned at U15. One 3 μm broad nerve extends antero-medially from the neuropil and branches laterally to form a dorsal and a ventral commissure at U12 and U9, respectively, together constituting an anterior nerve ring (anr, Figs. 5d, 6a-c, i, j and 9a, b). At the dorsal section of the anterior nerve ring (anr, Fig. 9), the acetylated α-tubulin-LIR is relatively weak, and the commissure consists of two transverse (anterior FMRF-amide-like-immunoreactive (LI-reactive) and posterior serotonin-LI-reactive) nerves, which eventually fuse dorso-laterally, forming the lateral sections of the anterior nerve ring. One anterior and one posterior short longitudinal nerves (cpn, Figs. 6b, i and 9b) extend from the ventral portion of the anterior nerve ring, innervating two median ciliary patches (described below). The neuropil supplies ventrally a pair of anterior ventro-median nerves (avmn, Figs. 6b, d, and 9b) extending between the anterior nerve ring and the post-pharyngeal ganglion posteriorly (pgg, Figs. 6b, f and 9b). It extends parallel to the pharyngeal median ciliated cell (pmcc, Fig. 10b, g), probably innervating it. Two pairs of dorso-median anterior nerves projections (danp, Figs. 6a, j and 9a) and two pairs of dorso-lateral anterior nerve projections (dlnp, Figs. 6a, j and 9a) extend from the anterior nerve ring and the lateral sides of the neuropil, respectively, projecting anteriorly. One pair of head lateral nerves (hln, Figs. 6a, b, e, j and 9a, b) extends from the lateral sides of the neuropil and bifurcates, posteriorly supplying a cell with a large and diffuse nucleus (possibly a gland cell (lgcb, Figs. 6a, e and 9a)), and anteriorly forming a nerve projection. Each of these anterior nerve projections probably innervates head sensory organs. Dorso-laterally, the posterior sides of the neuropil supply the ventro-lateral nerve cord (vlnc, Figs. 6b, c, d, f, g, j, k and 9b) of *D. aspetos*, which extends along the entire length of the specimen adjacent to the lateral longitudinal ciliary bands and the ventro-lateral longitudinal muscle, probably innervating these two structures. Two head dorso-posterior nerve (hdpn, Figs. 5e, 6a, h and 9a), extending along the pharynx, eventually supply the post-pharyngeal ganglion. They may innervate the anterior portion of the dorsal longitudinal muscle. A pair of head diagonal nerves (hdn, Figs. 6a, h and 9a) originates dorso-laterally of the neuropil, decussate dorsal to the pharynx at U22, and each extend ventro-laterally to a single perikaryon at U23. Comparison across specimens suggests that the position of these diagonal nerves corresponds to the position of the pharyngeal dorsal diagonal muscle (pddm, Figs. 2p and 3b, d), which it probably innervates. At U27 and U29, two thin nerves originate from the anterior ventro-median nerve, and form together at U28 a sub-pharyngeal commissure (spc, Figs. 6b, d and 9b). At U50, anterior to the testis, a thin trunk ventral commissure (tvc, Fig. 6g) is present. At U84, an anal ganglion (ang, Fig. 6k) of 6–8 cells supplies a pre-anal commissure (pac, Fig. 6k). Posterior to the anus, at U87, the two ventro-lateral nerve cords form the posterior commissure (pco, Fig. 6c, k), from which two nerve projections of the primary tube (nppt, Fig. 6c, k) extend.

#### Serotonin-like immunoreactivity (serotonin-LIR)

The nervous system shown by serotonin-LIR consists of a dorsal neuropil, the anterior nerve ring, anterior and posterior projections, the two ventro-lateral nerve cords, one posterior commissure as well as several perikarya.

Three main commissures (an anterior (sacn), a median (smcn) and a posterior (spcn)) in the brain neuropil show serotonin-LIR (Fig. 7a, d, e) as well as an isolated patch postero-median to the neuropil (spp, Fig. 7a, d, e). Three longitudinal nerves showing serotonin-LIR are found in the brain: i) the median-most brain nerve (smbn, Fig. 7a, d), ii) the paramedian brain nerve (spbn, Fig. 7a, d), and iii) the lateral brain nerve (slbn, Fig. 7a, d). Four very thin lateral nerves of the posterior commissure of the neuropil (slpn, Fig. 7a, d) form complex connections with the other nerves of the brain as well as to the dorso-median perikaryon (sdmp, Fig. 7a, c, d, e). A postero-lateral nerve node (spln, Fig. 7a, d, e) is present postero-laterally to the neuropil, being formed by the merging of several nerves, and supplies the ventro-lateral nerve cord (slnc, Fig. 7b, c, f). One dorso-lateral perikaryon (sdlp, Fig. 7a, c, d, e) supplies the postero-lateral nerve node, with a short nerve. The median-most brain nerve extends anteriorly from the posterior of the neuropil until U6 (Fig. 7a). The lateral brain nerve is short and extends from the postero-lateral nerve node, being supplied by some of the commissures of the neuropil (Fig. 7a). The paramedian brain nerve originates from the postero-lateral nerve node and supplies the serotonin-like-LI-reactive anterior nerve ring (sanr, Fig. 7a-d), subsequently extending more posteriorly as an anterior nerve projection until U4 (Fig. 7a, d). The anterior nerve ring consists dorsally of two and ventrally of one serotonin-LI-reactive nerves (sanr, Fig. 7a, b). The ventro-lateral nerve cord, consisting of two serotonergic-LI-reactive neurites, extends ventrally to supply a serotonin-LI-reactive para-pharyngeal cluster (sppc, Figs. 7b-e and 9b) with three perikarya, and extends to the posterior end forming the posterior commissure. Additionally, single serotonin-LI-reactive perikarya of the post-pharyngeal ganglion and of the anal ganglion are present respectively at U33 and U93 (spog, and spag, Fig. 7b, c, f) as well as nerve projections of the primary tube (snpt, Fig. 7c, f).

#### FMRF-amide-like immunoreactivity (FMRF-amide-LIR)

The FMRF-amide-LI-reactive nervous system consists of the brain neuropil, the anterior nerve ring, the anterior ventro-median nerve, the ventro-lateral nerve cord, the sub-pharyngeal commissure and the posterior commissure. Different parts of the nervous system show varying immunoreactivity intensities, as illustrated in Fig. 8.

The neuropil (fnp, Fig. 8a, c, d, f, g) consists of four connectives: two anterior and two posterior, supplied by several posterior and lateral perikarya. Antero-laterally to the neuropil, one pair of perikarya supplies a very short dorso-lateral anterior nerve projections (fpp, Fig. 8a, d, f, g) (corresponding to the base of the acetylated α-tubulin-LI-reactive projections (dlnp; Figs. 6a, j and 9a)). Additionally, three lateral perikarya of the brain (flpb, Fig. 8a, f, g) and a pair of dorso-posterior clusters of the brain (fdpc, Figs. 8a, f, g and 9a) with three perikarya, are present. Comparisons between differently stained specimens, and use of DAPI, enabled us to infer that the postero-median cell of the FMRF-amide-LI-reactive dorso-posterior cluster corresponds to the serotonin-LI-reactive dorso-posterior perikarya (sdmp, Fig.7; fdpc, Figs. 8a and 9a). Anteriorly, the neuropil supplies the anterior ventro-median nerve (fvmn, Figs. 8b, i and 9b), also supplied by two ventro-lateral perikarya of the brain (fvpb, Fig. 8a, e, i) and a ventral perikaryon of the anterior nerve ring (fvpr, Fig. 8b, i). The nerve ring (fanr, Fig. 8a-d, g, h, j) is supplied by one anterior and one posterior unpaired dorso-median perikarya (fpar, Fig. 8a, d, f, h). Ventro-posterior to the neuropil, a pair of tricellular clusters also supply the ventro-median nerve (fvnc, Fig. 8n, i), which extends further posterior until the two FMRF-amide-LI-reactive perikarya of the post-pharyngeal ganglion (fppg, Fig. 8b, c, k). The paired ventro-lateral nerve cords (flnc, Fig. 8b, c, i, k, l) is supplied by the postero-lateral part of the neuropil and by three anterior perikarya (fapn, Fig. 8b, c, e, i) (two anterior and one posterior, separated by 8 μm). The FMRF-amide-LI-reactive sub-pharyngeal commissure (fspc, Fig. 8b, i), ventro-lateral nerve cord, posterior commissure (fpco, Fig. 8c, l), and nerve projections of the primary tube (fnpt, Fig. 8c, l) follow the description of the acetylated α-tubulin-LIR.

### Ciliation

The locomotor ciliation consists of a dense ventro-anterior ciliated area and two thin ciliated bands, which are extending to the posterior part of the specimen at U87 (Fig. 10c). This general pattern supports the original description of Todaro et al. [17], although numerous details can be added. CLSM allowed the identification of individual multiciliated cells and determination of their precise pattern.

Dorsally, the muzzle is covered by two transverse rows of multiciliated cells. The anterior row consists of three pairs of relatively small head dorso-anterior ciliated cells (3 μm, hacc, Fig. 10a, e, f), while the posterior row is constituted by a pair of larger head postero-lateral dorsal ciliated cells (hpcc, Fig. CA,E) and a head dorso-median ciliated cell (hmcc, Fig. CA,E) of similar size (6 μm). The pattern of the head lateral ciliated cells (hlcc, Fig. 10a, f) could not be resolved in details due to the dorso-ventral mounting of the specimen. However, at least four cells at the dorso-lateral level are present, and probably the same number at the ventro-lateral level.

The ventral head bears 20 multiciliated cells organized in four paired longitudinal rows and one median row, containing 2,2,3,2,2,2,3,2,2 cells (Fig. 10b). Posterior to the head, ventral to the pharynx, from U9, only two adjacent rows of multiciliated cells are present on each side, containing one large pharyngeal median ciliated cell (pmcc, Fig. 10b, g, 45 μm long) and three pharyngeal lateral ciliated cells (plcc, Fig. 10b, g, h, 20–35 μm long), respectively. Posterior to the pharynx, only one paired lateral row of cells is present, which extends until the posterior trunk, as described originally [17].

The two ventro-median ciliary patches at U7 and U11 (acp, pcp, Fig. 10b, h, i, position of patches measured from the center) are innervated by two short diffuse 5 μm wide longitudinal nerves (cpn, Figs. 6b, i and 7b), joining perpendicularly the anterior nerve ring. Each patch also shows an acetylated α-tubulin-LI-reactive positive ring around the ciliated area. The divergent morphology of these anucleate multiciliated cells and their close relation to the nervous system suggest that they could be sensory structures.

Several sensoria are scattered along the body (ss, Fig. 10d, j), and two pairs of pharyngeal sensory cilia (mk and pk, Figs. 3a-d, i, j, 4c, g and 10i) are located in the pharyngeal region (see the nervous system section for further details).

Two pairs of nephridia are found along the body (Fig. 10d): the anterior pair is situated ventro-laterally and the posterior pair is located dorso-laterally, relatively close to the midline. The anterior pair of protonephridia (apn, Fig. 10d, j) is situated anterior to the testis at U42 and the cilia are 20 μm long. The posterior pair of protonephridia (ppn, Fig. 10d, k) is situated at U74 with 15 μm long cilia. Each nephridium seems to possess two straight coaxial cilia (c, c’, Fig. 10j, k), thus resembling the general paucitubulatinian protonephridia with its two adjacent monociliated terminal cells projecting into a non-ciliated canal cell and ending with a nephridiopore epidermal cell [2, 41]. The canal cell and the nephridiopore cell have not been stained, which is why information on the orientation and opening of protonephridia in *D. aspetos* is lacking.

## Discussion

### Phylogeny

The present phylogenetic analysis confirms that *Diuronotus aspetos* belongs to the monophyletic family Muselliferidae as proposed previously based on morphology [22, 23, 26]. We furthermore find Xenotrichulidae sister group to Muselliferidae (100 % support), opposed to its position next to Group B (“Chaetonotidae” + Dasytydae + Neogosseidae) in Kånneby et al. [26] (69 % PP). Moreover, the placement of *D. aspetos* considerably reduces the internal branch length of Muselliferidae, diminishing the possibility of long-branch attraction, with e.g. *Neodasys* ([49] and present study) or *Dactylopodola* [26], and the support of group B is now maximum. We further note two other interesting points: the sister group relationship between Neogosseidae and Dasydytidae is recovered [50], and the sister group relationship of marine *Aspidiophorus* to the remaining members of the Group B is found again, similarly to Kånneby et al. 2012 [49], but not Kånneby and Todaro [50].

### Musculature

**Fig. 11:**
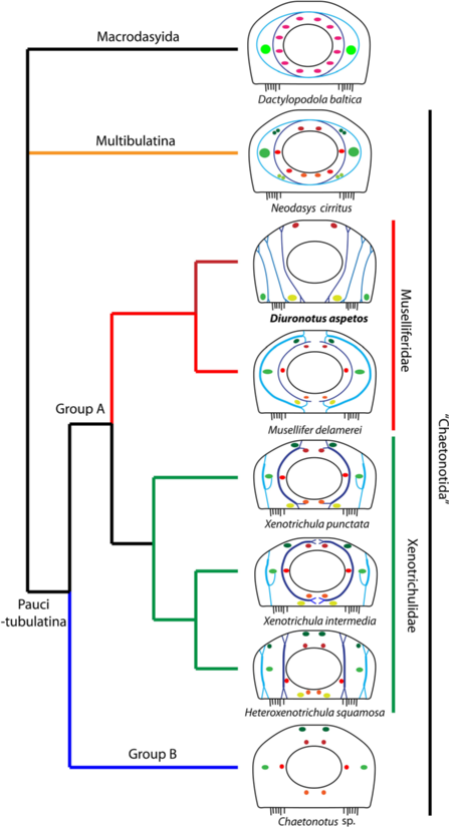
Evolution of musculature in Paucitubulatina. Each drawing presents the musculature as organized in a transverse section of the trunk with dorsal side pointing at the *top*. Adapted from [22], with addition of missing longitudinal muscles in the original figure, however, mentioned in the article. The musculature of *Neodasys cirritus* is modified from [59], and the phylogeny follows the results of the present study (Fig. 1). Possibly homologous muscles are illustrated with similar colors with the dorso-ventral muscles in *blue*, somatic longitudinal muscles in *green*, and splanchnic longitudinal muscles in *red*. See the discussion for further considerations on the different homologies

The overall musculature of *Diuronotus aspetos* is relatively simple, consisting of only three pairs of longitudinal muscles in the trunk as well as a unique arrangement of multiple dorso-ventral muscles. The number of pairs of longitudinal muscles in *D. aspetos* is inferior to what is found in most Paucitubulatina, having at least five, often six, pairs of longitudinal muscles, that are often distributed as three pairs of splanchnic and three pairs of somatic muscles (*Musellifer*, *Draculiciteria*, *Heteroxenotrichula*, *Xenotrichula*, *Chaetonotus*, *Aspidiophorus*, and *Polymerurus*) [22, 31, 32] (Fig. 11). The previously proposed hypothetical ancestral state of musculature in Paucitubulatina [2] shows a split of the dorsal longitudinal muscle (musculus dorsalis) into two branches, not present in *D. aspetos* that instead has a branch of the ventral longitudinal muscle running dorsally. The more complex branching pattern of the ventral longitudinal muscle may be an adaptation to the large size of *D. aspetos*, compensating for the low number of longitudinal muscle.

The helicoidal musculature encircles the dorsal longitudinal muscles but not the ventral longitudinal muscles or the ventro-lateral longitudinal muscles. The relative position of the dorsal longitudinal muscle indicates a homology to the dorsal splanchnic muscle of other Paucitubulatina (Fig. 11) (see Kieneke and Schmidt-Rhaesa [2] for further discussion and limitations of this notion). However, its more dorsal position indicates that it supports the body wall rather than the digestive tract, perhaps furthermore compensating for the missing dorso-dermal muscle branch in *D. aspetos*. The ventro-lateral longitudinal muscle of *D. aspetos* can be homologized with the somatic ventro-lateral muscle (or musculus lateralis) of other Gastrotricha, and the ventral longitudinal muscle resembles those found in the paucitubulatinian *Musellifer delamerei*, *Xenotrichula intermedia* Remane [57] and *Heteroxenotrichula squamosa* Wilke, 1954 [22, 58] (Fig. 11).

The unique semi-circular muscles of *D. aspetos* may aid the oviposition together with the dorso-ventral muscles, hereby functionally replacing the dorso-dermal longitudinal muscle split enveloping the egg in other Paucitubulatina [22]. They likely act as the posterior complete circular muscles found in the region of the sexual organs of *Neodasys* cf. *uchidai* Remane, 1961 [59, 60]. Functionally similar circular muscles are also found in the meiofauna gnathiferan *Gnathostomula armata* Riedl [61] and *Gnathostomula peregrina* Kristeuer [62] (Gnathostomulida), here arranged in dense pattern around the posterior male organs [63, 64].

The evolution of the dorso-ventral muscles of Chaetonotida as deriving from the circular musculature has been one of the central debates in previous studies [22, 31]. In Macrodasyida and Multitubulatina, the circular musculature consists of splanchnic and somatic elements, the former encircling the intestine and the latter, which derives from splanchnic elements, encircles the ventro-lateral longitudinal muscle on both sides [2, 22, 59]. In Paucitubulatina, the trunk circular muscles are either absent or have a dorso-ventral orientation (i.e. incomplete circular muscles). Such dorso-ventral muscles are found in Xenotrichulidae and Muselliferidae (group A, Fig. 1) [2, 22, 31, 65] (Fig. 11). Hence, if the group A is monophyletic, it means that this arrangement is a synapomorphy of this group, and that the group B lost the dorso-ventral, or circular, muscles (regained in the genus *Polymerurus* [32]). Comparatively, *D. aspetos* is the only Paucitubulatina with more than two sets of dorso-ventral muscles in the transverse axis. The median-most dorso-ventral muscles may be homologous with the visceral circular muscles in other gastrotrichs. The lateral sets of dorso-ventral muscles present varying positions relative to the longitudinal muscles, making homologies difficult to assess. Furthermore, no dorso-ventral muscles are present lateral to the ventro-lateral longitudinal muscle, which would be an arrangement expected from a derived somatic semi-circular muscle such as found in other Paucitubulatina [20, 22], and see Fig. 11. Consequently, the inner-most dorso-ventral muscles of *D. aspetos* can be homologized with the semi-circular muscles of other Paucitubulatina (Fig. 11), and the arrangement of the other dorso ventral muscles is so far unique in Gastrotricha [2, 22, 32].

The head diagonal muscle of *D. aspetos* may be homologous to the head semi-circular muscle found in *Musellifer delamerei* and *Dactylopodola baltica* (Remane, 1926) [22, 66, 67] showing the same anterior position and shape though a different orientation. It is however probably only homologous to the one found in *Musellifer* since it is the sister group of *Diuronotus*, while *Dactylopodola* is a distantly related Macrodasyida (Fig. 1). The posterior diagonal muscle and the diagonal muscle of the tubes resemble a muscle found in the posterior region of *Heteroxenotrichula squamosa* (Fig. 3a, [22]), but no similar muscle exist in *Musellifer delamerei*, *Xenotrichula intermedia* or *X. punctata* Wilke, 1954. The lack of similar muscle in the closely related taxa within group A indicates that this muscle is an apomorphy of *Diuronotus* (Fig. 11) [22, 58]. The so-called cross-over muscles found in Macrodasyida with a bilobed caudal end has a similar function, being involved in the movement of the posterior tubes, yet with the lack of presence in other Paucitubulatina and different morphology in *D. aspetos* it is most likely of convergent origin [2, 66, 68].

### Nervous system

To date, *Xenotrichula intermedia* and *Xenotrichula velox* Remane [69] are the only other Paucitubulatina for which the nervous system has been studied with CLSM [13], therefore the present study adds valuable information. On the other hand, the nervous system of *Neodasys* (Multitubulatina was described in details with CLSM) [12, 39], as well as several Macrodasyida [15, 35, 36, 38, 39, 70, 71]. Furthermore *Cephalodasys maximus* Remane [67] and *Turbanella cornuta* Remane [72] have been described in detail with TEM [37, 73]. This offers a broad, but not comprehensive, literature for comparing the nervous system of *D. aspetos* with other Gastrotricha.

#### Stomatogastric nervous system

Similar to other Chaetonotida [12, 13, 18], one dorso-median and two ventro-lateral longitudinal nerves constitute the overall pharyngeal nervous system of *Diuronotus aspetos*. However, the present study finds several additional structures previously undescribed for chaetonotids such as: i) five additional symmetric and one asymmetric longitudinal nerves branching off from the main nerves, ii) two previously undescribed commissures (anterior-most buccal nerve ring, middle pharyngeal nerve ring), and iii) a pair of ventro-lateral pharyngeal ganglia (innervating anterior sensory structures).

However, only the pharyngeal nervous system of *Cephalodasys maximus* has been comprehensively described [37] and little is known about the pharyngeal nervous system of Chaetonotida (but see [12, 13]). Nonetheless, ultrastructural studies by Teuchert (1877) [73] and Ruppert [18] provide various details of the pharynx in several gastrotrichs, including some details on *Diuronotus* sp.

In Macrodasyida, the inverted organization of the pharynx generally offers one ventro-median and two dorso-lateral nerves as well as one additional dorso-median nerve [18]. In *Turbanella cornuta*, an additional asymmetric “thick” ventro-lateral nerve is also present in the pharynx [73], which resembles the one short asymmetric dorso-lateral longitudinal nerve found in *D. aspetos* to a certain degree. *Cephalodasys maximus* presents a pair of ventro-lateral asymmetric nerves (one short, one long) in the pharynx, but they originate more posteriorly from the pharyngeal nerve ring [37]. The probable convergent origin of the asymmetric pharyngeal nerves in the morphologically and phylogenetically diverse Macrodasyida *C. maximus* (Cephalodasyidae) and *T. cornuta* (Turbanellidae) contradicts their homology with the one found in *D. aspetos*, but shows that asymmetry in the pharynx of gastrotrichs might have evolved multiple times in Gastrotricha [15, 29].

Macrodasyida and *Neodasys* possess multiple triplets of pharyngeal cilia [18], which according to the basal interrelationship of Gastrotricha suggest that the lack of reports of these structures in Paucitubulatina represents a loss (rather than a primary absence). A single short pair may have either re-appeared in *Diuronotus* (according the topology of our tree (Fig. 1)) or alternatively have been overlooked in previous studies of the sister group Musellifer, or the other members of the group A (Fig. 1), Xenotrichulidae. Ruppert [18] also discusses the presence of discrete glands opening in the mouth of *Chaetonotus* and *Musellifer*, possibly homologous to the anterior pharyngeal glands here described for *D. aspetos*.

Herein is further revealed a presently undescribed pharyngeal canal system within the musculature, occasionally lined by nerves. However, a single transverse TEM micrograph of *Diuronotus* sp. by Ruppert (Figure 14, [18]), purportedly from the level of the ventro-lateral pharyngeal ganglion, reveals a dorso-lateral as well as three ventro-lateral electron-lucent areas, which most likely resemble the canal system. The system may be unique to *Diuronotus* or Muselliferidae, and its function is unknown.

#### Central nervous system

**Fig. 12:**
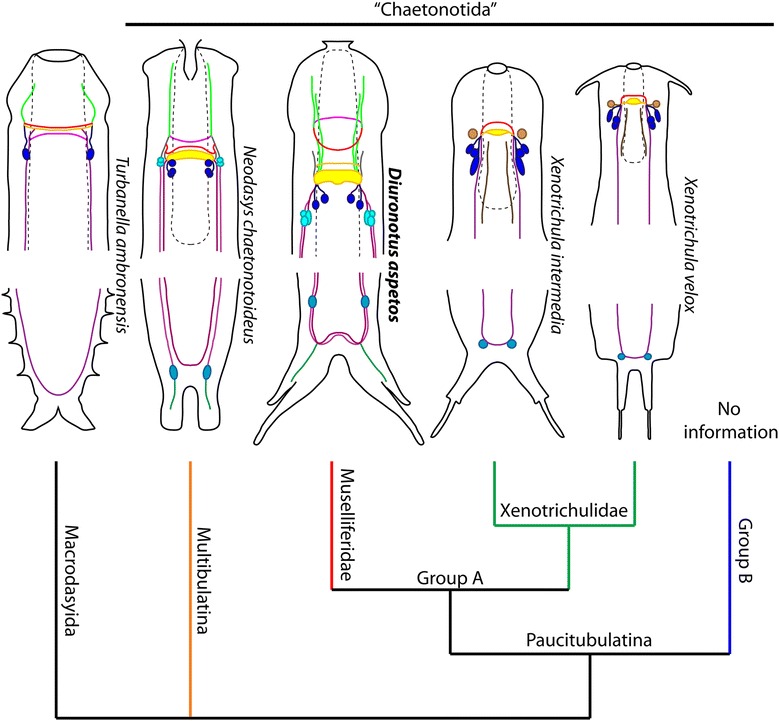
Evolution of the serotonin-LI-reactive nervous system in Chaetonotida. Schematic presentation of dorsal view, anterior end on *top*, posterior end on *bottom*. *Turbanella ambronensis* adapted from [71], *Neodasys chaetonotoideus* adapted from [12], and *Xenotrichula intermedia* and *X. velox* adapted from [13]. If CLSM pictures are available for representatives of the Group B [77], no formal descriptions exist for this group. The phylogeny follows the results of the present study (Fig. 1). See the discussion for further considerations on the different homologies, and variation between the different immunostainings

The overall morphology of the nervous system of *Diuronotus aspetos* is similar to other gastrotrichs [2] consisting of a “dumbbell-shaped” dorsal brain with a dorsal neuropil and a pair of ventro-lateral nerves. However, additional nerves and specific perikarya are found in *D. aspetos*. A summary figure (Fig. 12) shows the evolution of the serotonin-LI-reactive nervous system in Chaetonotida, since this is the most comparable immunostaining across Gastrotricha.

##### Longitudinal nerves

Anterior to the brain neuropil of *Diuronotus aspetos*, four pairs of dorsal nerve projections are found (acetylated α-tubulin-LI-reactive, in addition to several minor neurites left undescribed), most likely related to the anterior sensoria. Similar nerve projections are described in *Neodasys chaetonotoideus* Remane, 1927 [12, 74], *Cephalodasys maximus* [37] and *Thaidasys tongiorgii* Todaro et al. [15] but due to the scarcity of these descriptions, a closer homology cannot yet be stated. Another two pairs of nerves project antero-ventrally from the brain (serotonin-LI-reactive) in *D. aspetos*, one of which may be homologous to the commonly found single pair of serotonin-LI-reactive ventral projections in other gastrotrichs (e.g. *Neodasys chaetonotoideus*, *Dactylopodola* or *Oregodasys cirratus* [12, 36, 38]) (Fig. 12). A similar positioned pair of FMRF-amide-LI-reactive projections is present in *Lepidodasys worsaae* Hochberg and Atherton [70] and *Xenotrichula* [13, 70], and in *Oregodasys cirratus* these are expressing both FMRF-amide-LIR and serotonin-LIR [38], suggesting that the neurotransmitters of these nerves can vary, and that they are a general character of Gastrotricha (cf. nervous system drawing in [2]).

Another striking character found in *D. aspetos* is the paired anterior ventro-median nerve in the anterior trunk. Short, paired anterior ventro-median nerves are also found in *Thaidasys tongiorgii*, *Turbanella* cf. *hyalina* Schultze [75], and extending the entire body length in *Oregodasys cirratus* [15, 38, 39]. However, the exact connection to other nerves and their extension differs from those of *D. aspetos*. Moreover, studies on *Neodasys chaetonotoideus* and on the closely related *Xenotrichula* [12, 13] did not find similar paired anterior ventro-median nerves and we therefore consider the ventro-median nerves in *D. aspetos* a convergence related to the different ciliation of this species.

The paired short dorso-lateral nerves in *D. aspetos* (hdpn, Figs. 6a, h and 9a) are similar in position and extension to the paired dorsal nerves described in the distantly related Macrodasyida *C. maximus* [37] as well as the dorsal pharyngeal fibers found in the phylogenetically close *Xenotrichula* [13] (and Fig. 1, with Muselliferidae sister group of Xenotrichulidae), of which the latter at least seems to be homologous to the dorsal nerves of *D. aspetos*.

##### Ganglia and perikarya

Several immunoreactive perikarya can be compared to other gastrotrichs, mostly *Neodasys* and *Xenotrichula*. However, immunoreactivity of the perikarya is quite variable, and only a fraction of the brain cells are immunoreactive.

Only five pairs of serotonin-LI-reactive perikarya are found in the brain of *Diuronotus aspetos*, situated postero-laterally to the neuropil. They comprise two dorsal pairs of perikarya, supplying the neuropil, and a ventral pair of para-pharyngeal clusters (spgg, Figs. 7b-e and 9b) with three perikarya each, supplying the ventro-lateral nerve cords. The closely related *Xenotrichula* does not possess a serotonin-LI-reactive equivalent to the ventral clusters, but possesses four dorsal pairs of serotonin-LI-reactive perikarya [13], two of which are likely homologous to the two dorsal pairs found in *D. aspetos* (Fig. 12). *Neodasys chaetonotoideus* possesses three dorso-lateral serotonin-LI-reactive perikarya, which have similar positions and connection to the neuropil than the dorsal serotonin-LI-reactive perikarya of *D. aspetos.* Moreover, *N. chaetonotoideus* possesses a similar paired cluster of para-pharyngeal serotonin-LI-reactive perikarya, associated to the ventro-lateral nerve cords. This suggests that *N. chaetonotoideus* and *D. aspetos* might share some plesiomorphic traits of their serotonin-LI-reactive nervous system, whereas *Xenotrichula* represents a derived condition (Fig. 12). Depending on the position of *Neodasys the* para-pharyngeal cluster could be either a synapomorphy of Chaetonotida or Gastrotricha. In the Macrodasyida investigated to date, the serotonin-LI-reactive brain is generally simpler than in Chaetonotida, comprising only one dorsal commissure and one pair of dorso-lateral perikarya [15, 38, 71] (sometimes two [76]) (Fig. 12), although additional serotonin-LI-reactive perikarya can be found in *Dactylopodola* [36], and *Paradasys subterraneus* Remane [57] (personal observations).

The FMRF-amide-LI-reactive perikarya of the brain of *D. aspetos* are numerous (at least 16 paired and two unpaired of various intensity of the immunoreactivity) and surround the brain neuropil dorsally, ventrally and laterally. Due to the high number and variation of FMRF-amide-LIR of the brain in Gastrotricha, we limit our comparison of *D. aspetos* to the closely related *Xenotrichula* [13]. Homologies of the perikarya depend on whether the anterior dorsal and ventral FMRF-amide-LI-reactive commissures in *Xenotrichula* are homologous to the anterior nerve ring of *D. aspetos*. If so, the two dorso-median FMRF-amide-LI-reactive perikarya found connected to the anterior dorsal commissure in *Xenotrichula*, may be homologous to the two dorso-median found in *D. aspetos*. The two additional described paired lateral and ventral FMRF-amide-LI-reactive perikarya in *Xenotrichula* are difficult to homologize with those of *D. aspetos*. However, one pair of undescribed ventral FMRF-amide-LI-reactive perikarya is found laterally on the ventral commissure of *Xenotrichula* (Figure 4h, [13]) and is possibly homologous to the ventral perikarya of the FMRF-amide-LI-reactive nerve ring in *D. aspetos*. Finally, one of the cells of the FMRF-amide-LI-reactive dorso-posterior cluster of the brain of *D. aspetos* may be homologous to the single pair of perikarya found in *Xenotrichula* in the same position.

In a position similar to the post-pharyngeal ganglion of *D. aspetos*, two pairs of FMRF-amide-LI-reactive (no serotonin-LIR) perikarya are described supplying the ventro-lateral nerve cord in *Xenotrichula* [13]. Between these two pairs, two short transverse FMRF-amide-LI-reactive neurites almost constitute a commissure similar to the one of *D. aspetos,* suggesting that the posterior-most pair of perikarya in *Xenotrichula* is homologous to the ganglia found in *D. aspetos*.

An anal pair of serotonin-LI-reactive perikarya contained in the anal ganglion is found in *D. aspetos* as well as a posterior commissure, similar to what is described for *Xenotrichula* and *Neodasys chaetonotoideus* [12, 13]. Yet, no equivalent is found in any Macrodasyida (Fig. 12), confirming that it is either a synapomorphy of Chaetonotida or Gastrotricha [2], depending of the phylogenetic position of *Neodasys*. Herein observations show that the anal ganglion consists of several cells in *D. aspetos*, contrary to other Chaetonotida [77]. Moreover, we describe an additional pre-anal commissure, originating at the anal ganglion, only revealed by acetylated α-tubulin-LIR and hitherto not found in other gastrotrichs.

##### Brain commissures

*Diuronotus aspetos* does not show a commissure situated directly ventrally to the main brain neuropil contrary to most Gastrotricha documented, including Chaetonotida (e.g. [12, 13, 15, 39]). This character was central in previous discussions on a possible close relationship between Cycloneuralia and Gastrotricha (e.g. [36, 39, 78, 79]), rejected today (e.g. [3, 80]) since, recent interpretations of the brain of Gastrotricha show that it is not truly circular [80]. In *D. aspetos*, the anterior nerve ring is associated to the brain and its ventral portion resembles the ventral brain commissure of other Gastrotricha, although being more anterior (Fig. 12). Furthermore, *Xenotrichula* possesses one ventral FMRF-amide-LI-reactive commissure anterior of the brain [13]. If the FMRF-amide-LI-reactive anterior commissure of the brain and ventral commissure of *Xenotrichula* are continuous, it can be speculated that *Xenotrichula* also possesses an anterior nerve ring, and therefore this arrangement might be a synapomorphy of the group A (Fig. 1).

### Ventral ciliation

The main difference from the original description is that the head ventral ciliation forms two medially separated ciliated areas in *Diuronotus aspetos*. Furthermore, a more detailed pattern has been deduced, showing the relevance of CLSM for determining ciliary arrangement [81–83] (but see also Kerbl et al., in prep; Bekkouche and Worsaae, in prep, respectively on Dinophilidae (Annelida) and Micrognathozoa). This also opens the way to a new kind of characters in interstitial animals, which could have a systematics value: the pattern of the multi-ciliated cells. Indeed, preliminary results showing variation in the pattern of the ventral multi-ciliated cells of Thaumastodermatidae support this idea (Bekkouche and Worsaae unpublished). Unfortunately, though the description of the general pattern of the ventral ciliation is common in Chaetonotida, details are rare. In few cases, more details were given, for instance for Neogosseidae (with exact description of the ciliary bands [84]). A few studies have described ciliary patches in the head of some Chaetonotidae (e.g. [85, 86]), but no precise information on the cells themselves has been given, which is why it is unknown whether each patch or band is composed of one or several cells. This limitation of light and electron microscopy can be overcome by employment of CLSM, but due to the lack of similar studies, we cannot yet comment on the evolution of the fine detailed ciliation pattern of Gastrotricha.

Interestingly, some Paucitubulatina show unpaired ciliary patches on the ventral midline of the head, e.g. *Halichaetonotus atlanticus*, Kisielewski [85], *Arenotus strixinoi* Kisielewski [86] or *Kijanebalola devestiva* Todaro et al. [84], but details are insufficient to hypothesize any homology with the ventro-median ciliary patches of *D. aspetos*.

### Protonephridial system

Until the present study, all three previously studied Paucitubulatina were known to possess only one pair of protonephridia (*Xenotrichula carolinensis stylensis* Mock [87], *Chaetonotus maximus* Ehrenberg, 1831 [41, 88] and *Polymerurus nodicaudus* (Voigt, 1901) [42, 89]). In this context, *Diuronotus aspetos* is the only Paucitubulatina known to have more than one pair of protonephridia, suggesting, according our phylogeny (Fig. 1), an addition of a pair of protonephridia. However, studies on the protonephridial system of *Musellifer* are needed to confirm if the presence of a single pair of protonephridia has a phylogenetic value or is due to size dependency. Indeed, the number of pairs of protonephridia in other Gastrotricha is variable and seems to be roughly size dependent (e.g. two pairs for the ca. 250 μm long *Dactylopodola baltica,* and 11 pairs for the ca. 1 mm long *Mesodasys laticaudatus* Remane, 1951 [90, 91]).

## Conclusion

The present study is the first detailed anatomical description of a member of Muselliferidae, and only the second description of the nervous system within the larger clade Paucitubulitina within Gastrotricha [13]. The key phylogenetic position of *Diuronotus* in Gastrotricha, the newly discovered traits of the nervous, muscular and ciliary system, and the comparison to phylogenetically related lineages reported here lead to new hypotheses on nervous trait homologies and evolution. Our results provide an important step towards understanding the evolution of organ systems within Gastrotricha, and promise new insights for detailed anatomical studies of the major organ systems.

The musculature of *D. aspetos* presents unique traits for Paucitubulatina (reduction of the number of longitudinal muscles, the addition of dorso-ventral muscles) and previously undescribed muscles, showing that the musculature is more varied than expected in Paucitubulatina [22].

Although the nervous system of *D. aspetos* is similar to other gastrotrichs overall, detailed studies revealed numerous unknown minor components of the gastrotrich nervous system (e.g. one pair of anterior ventro-median nerves, one pair of dorso-posterior nerves, two pairs of additional ganglia). This indicates that similar detailed immunohistochemical studies on other species may similarly reveal new elements in the diversity of the gastrotrich nervous system and highlight additional homologies. This is supported by the multiple similarities uncovered from comparison with the immunohistochemistry studies of the closely related *Neodasys* and *Xenotrichula*, suggesting, e.g., homologous brain commissures and perikarya.

The pharynx displays an intriguing canal system and nerves, so far undescribed in Gastrotricha. Additionally, investigation of the ventral ciliation reveals details of the cellular arrangement which refines the previous description [17] and may show to be of broader systematic importance within Gastrotricha.

The many new discoveries in the different organ systems of Gastrotricha unravel an intriguing hidden diversity of morphological traits in Gastrotricha. This immediately stresses the importance of similar studies on additional key lineages of Paucitubulatina such as *Musellifer*, *Draculiciteria*, and marine *Aspidiophorus*, as well as freshwater “Chaetonotidae,” in order to complement this picture and address the surprisingly complex evolution of gastrotrich morphology.

## References

[1] Edgecombe GD, Giribet G, Dunn CW, Hejnol A, Kristensen RM, Neves RC, Rouse GW, Worsaae K, Sørensen MV (2011). Higher-level metazoan relationships: recent progress and remaining questions. Organ Divers Evol.

[2] Kieneke A, Schmidt-Rhaesa A. 1. Gastrotricha. In: Schmidt-Rhaesa A, Editor. Handbook of Zoology, Gastrotricha and Gnathifera. vol. 3. Berlin/Munich/Boston: De Gruyer; 2015.

[3] Laumer CE, Bekkouche N, Kerb A, Goetz F, Neves RC, Sorensen MV, Kristensen RM, Hejno A, Dunn CW, Giribet G (2015). Spiralian phylogeny informs the evolution of microscopic lineages. Curr Biol.

[4] Hyman LH (1951). The Invertebrates. Vol. 3, Acanthocephala, aschelminthes, and entoprocta : the pseudocoelomate bilateria.

[5] Bütschli O. Untersuchungen über freilebende Nematoden und die Gattung Chaetonotus. Zeitschrift für wissenschaftliche Zoologie. 1876; 363–413.

[6] Rieger RM (1976). Monociliated epidermal cells in Gastrotricha; significance for concepts of early metazoan evolution. Zeitschrift Zool Syst EvolForsch.

[7] Zrzavý J, Mihulka S, Kepka P, Bezdek A, Tietz D (1998). Phylogeny of the Metazoa based on morphological and 18S ribosomal DNA evidence. Cladistics.

[8] Cavalier-Smith T (1998). A revised six-kingdom system of life. Biol Rev Camb Philos Soc.

[9] Giribet G (2008). Assembling the lophotrochozoan (=spiralian) tree of life. Philos Trans R Soc B Biol Sci.

[10] Struck TH, Wey-Fabrizius AR, Golombek A, Hering L, Weigert A, Bleidorn C, Klebow S, Iakovenko N, Hausdorf B, Petersen M (2014). Platyzoan paraphyly based on phylogenomic data supports a noncoelomate ancestry of spiralia. Mol Biol Evol.

[11] Kieneke A, Arbizu PM, Riemann O (2008). Body musculature of *Stylochaeta scirtetica* Brunson, 1950 and *Dasydytes* (*Setodytes*) *tongiorgii* (Balsamo, 1982) (Gastrotiricha : Dasydytidae): A functional approach. Zool Anz.

[12] Rothe BH, Schmidt-Rhaesa A, Kieneke A (2011). The nervous system of *Neodasys chaetonotoideus* (Gastrotricha: Neodasys) revealed by combining confocal laserscanning and transmission electron microscopy: evolutionary comparison of neuroanatomy within the Gastrotricha and basal Protostomia. Zoomorphology.

[13] Rothe BH, Kieneke A, Schmidt-Rhaesa A (2011). The nervous system of *Xenotrichula intermedia* and *X. velox* (Gastrotricha: Paucitubulatina) by means of immunohistochemistry (IHC) and TEM. Meiofauna Marina.

[14] Todaro MA, Leasi F, Hochberg R (2014). A new species, genus and family of marine Gastrotricha from Jamaica, with a phylogenetic analysis of Macrodasyida based on molecular data. Syst Biodivers.

[15] Todaro MA, Dal Zotto M, Leasi F (2015). An Integrated Morphological and Molecular Approach to the Description and Systematisation of a Novel Genus and Species of Macrodasyida (Gastrotricha). PLoS ONE.

[16] Kolicka M, Dabert M, Dabert J, Kånneby T, Kisielewski J. *Bifidochaetus*, a new Arctic genus of freshwater Chaetonotida (Gastrotricha) from Spitsbergen revealed by an integrative taxonomic approach. Invertebrates Systematics. 2016. (in press).

[17] Todaro MA, Balsamo M, Kristensen RM (2005). A new genus of marine chaetonotids (Gastrotricha), with a description of two new species from Greenland and Denmark. J Mar Biol Assoc U K.

[18] Ruppert EE (1982). Comparative ultrastructure of the gastrotrich pharynx and the evolution of myoepithelial foreguts in aschelminthes. Zoomorphology.

[19] Ruppert EE (1988). Introduction to the study of meiofauna.

[20] Ruppert EE, Harrison FW, Ruppert EE (1991). Gastrotricha. Microscopic anatomy of invertebrates Volume 4: Aschelminthes.

[21] Kieneke A (2015). Record of the ‘Arctic’ marine gastrotrich *Diuronotus aspetos* (Paucitubulatina) from the southern North Sea. Mar Biodivers.

[22] Leasi F, Todaro MA (2008). The muscular system of *Musellifer delamarei* (Renaud-Mornant, 1968) and other chaetonotidans with implications for the phylogeny and systematization of the Paucitubulatina (Gastrotricha). Biol J Linn Soc.

[23] Balsamo M, Guidi L, Ferraguti M, Pierboni L, Kristensen RM (2010). *Diuronotus aspetos* (Gastrotricha): new morphological data and description of the spermatozoon. Helgol Mar Res.

[24] D’Hondt JL, Barnes H (1971). Gastrotricha. Oceanography and Marine Biology: An Annual Review.

[25] Hochberg R, Litvaitis MK (2000). Phylogeny of Gastrotricha: a morphology-based framework of gastrotrich relationships. Biol Bull.

[26] Kånneby T, Atherton S, Hochberg R (2014). Two new species of *Musellifer* (Gastrotricha: Chaetonotida) from Florida and Tobago and the systematic placement of the genus within Paucitubulatina. Mar Biol Res.

[27] Todaro MA, Telford MJ, Lockyer AE, Littlewood DTJ (2006). Interrelationships of the Gastrotricha and their place among the Metazoa inferred from 18S rRNA genes. Zool Scr.

[28] Paps J, Riutort M (2012). Molecular phylogeny of the phylum Gastrotricha: New data brings together molecules and morphology. Mol Phylogenet Evol.

[29] Kieneke A, Riemann O, Ahlrichs WH (2008). Novel implications for the basal internal relationships of Gastrotricha revealed by an analysis of morphological characters. Zool Scr.

[30] Hummon WD (1969). *Musellifer sublitoralis* a New Genus and Species of Gastrotricha from San Juan Archipelago Washington. Trans Am Microsc Soc.

[31] Hochberg R, Litvaitis MK (2001). The musculature of *Draculiciteria tessalata* (Chaetonotida, Paucitubulatina): implications for the evolution of dorsoventral muscles in Gastrotricha. Hydrobiologia.

[32] Leasi F, Rothe BH, Schmidt-Rhaesa A, Todaro MA (2006). The musculature of three species of gastrotrichs surveyed with confocal laser scanning microscopy (CLSM). Acta Zool.

[33] Kieneke A, Ostmann A (2012). Structure, function and evolution of somatic musculature in Dasydytidae (Paucitubulatina, Gastrotricha). Zoomorphology.

[34] Hochberg R, Litvaitis MK (2001). A muscular double helix in gastrotricha. Zool Anz.

[35] Hochberg R, Litvaitis MK (2003). Ultrastructural and immunocytochemical observations of the nervous systems of three macrodasyidan gastrotrichs. Acta Zool.

[36] Rothe BH, Schmidt-Rhaesa A (2009). Architecture of the nervous system in two *Dactylopodola* species (Gastrotricha, Macrodasyida). Zoomorphology.

[37] Wiedermann A (1995). On the ultrastructure of the nervous system in *Cephalodasys maximus* (Macrodasyida, Gastrotricha). Zur Ultrastruktur des Nervensystems bei *Cephalodasys maximus* (Macrodasyida, Gastrotricha). Microfauna Marina.

[38] Rothe BH, Schmidt-Rhaesa A (2010). *Oregodasys cirratus*, a new species of Gastrotricha (Macrodasyida) from Tenerife (Canary Islands), with a description of the muscular and nervous system. Meiofauna Marina.

[39] Hochberg R (2007). Comparative immunohistochemistry of the cerebral ganglion in Gastrotricha: an analysis of FMRFamide-like immunoreactivity in *Neodasys cirritus* (Chaetonotida), *Xenodasys riedli* and *Turbanella* cf. *hyalina* (Macrodasyida). Zoomorphology.

[40] Schöpfer-Sterrer C (1969). *Chordodasys riedli* gen. nov. spec. nov. a macrodasyoid gastrotrich with a chordoid organ. Cah Biol Mar.

[41] Kieneke A, Ahlrichs WH, Arbizu PM, Bartolomaeus T (2008). Ultrastructure of protonephridia in *Xenotrichula carolinensis syltensis* and *Chaetonotus maximus* (Gastrotricha : Chaetonotida): comparative evaluation of the gastrotrich excretory organs. Zoomorphology.

[42] Kieneke A, Hochberg R (2012). Ultrastructural observations of the protonephridia of *Polymerurus nodicaudus* (Gastrotricha: Paucitubulatina). Acta Zool.

[43] Meyer CP (2003). Molecular systematics of cowries (Gastropoda: Cypraeidae) and diversification patterns in the tropics. Biol J Linn Soc.

[44] Giribet G, Carranza S, Baguna J, Riutort M, Ribera C (1996). First molecular evidence for the existence of a Tardigrada + Arthropoda clade. Mol Biol Evol.

[45] Brown S, Rouse G, Hutchings P, Colgan D (1999). Assessing the usefulness of histone H3, U2 snRNA and 28S rDNA in analyses of polychaete relationships. Aust J Zool.

[46] Vonnemann V, Schrodl M, Klussmann-Kolb A, Wagele H (2005). Reconstruction of the phylogeny of the Opisthobranchia (Mollusca : Gastropoda) by means of 18S and 28S rRNA gene sequences. J Molluscan Stud.

[47] Hall TA (1999). BioEdit: a user-friendly biological sequence alignment editor and analysis program for Windows 95/98/NT. Nucleic Acids Symp Ser.

[48] Geer LY, Marchler-Bauer A, Geer RC, Han L, He J, He S, Liu C, Shi W, Bryant SH (2010). The NCBI BioSystems database. Nucleic Acids Res.

[49] Kånneby T, Todaro MA, Jondelius U (2013). Phylogeny of Chaetonotidae and other Paucitubulatina (Gastrotricha: Chaetonotida) and the colonization of aquatic ecosystems. Zool Scr.

[50] Kånneby T, Todaro MA (2015). The phylogenetic position of Neogosseidae (Gastrotricha: Chaetonotida) and the origin of planktonic Gastrotricha. Organ Divers Evol.

[51] Todaro MA, Kånneby T, Dal Zotto M, Jondelius U (2011). Phylogeny of thaumastodermatidae (Gastrotricha: Macrodasyida) inferred from nuclear and mitochondrial sequence data. PLoS ONE.

[52] Vaidya G, Lohman DJ, Meier R (2011). SequenceMatrix: concatenation software for the fast assembly of multi-gene datasets with character set and codon information. Cladistics.

[53] Huelsenbeck JP, Ronquist F (2001). MRBAYES: Bayesian inference of phylogeny. Bioinformatics.

[54] Rambaut A, Suchard MA, Xie D, Drummond AJ. 2014. Tracer v1.6, Available from http://beast.bio.ed.ac.uk/Tracer. Accessed 23 Aug 2016

[55] Hummon WD, Balsamo M, Todaro MA (1992). Italian Marine Gastrotricha 1. 6 New and One Redescribed Species of Chaetonotida. Bollettino Di Zoologia.

[56] Renaud-Mornant J (1968). Présence du genre *Polymerurus* en milieu marin, description de deux espèces nouvelles (Gastrotricha, Chaetonotoidae). Pubblicazioni della Stazione Zoologica di Napoli.

[57] Remane A (1934). Die Gastrotrichen des Küstengrundwassers von Schilksee. Schriften des Naturwissenschaftlichen Vereins für Schleswig-Holstein.

[58] Wilke U (1954). Mediterrane Gastrotrichen. Zoologische Jahrbücher.

[59] Hochberg R (2005). Musculature of the primitive gastrotrich *Neodasys* (Chaetonotida): functional adaptations to the interstitial environment and phylogenetic significance. Marine Biology (Berlin).

[60] Remane A (1961). *Neodasys uchdai* nov. spec., eine zwiete *Neodasys* Art (Gastrotrich Chaetoidea). Kiel Meeresforsch.

[61] Riedl R (1971). On the Genus *Gnathostomula* (Gnathostomulida). Int Rev Hydrobiol.

[62] Kristeuer E (1969). On some species of Gnathostomulida from Bimini, Bahamas.

[63] Müller MCM, Sterrer W (2004). Musculature and nervous system of *Gnathostomula peregrina* (Gnathostomulida) shown by phalloidin labeling, immunohistochemistry, and cLSM, and their phylogenetic significance. Zoomorphology (Berlin).

[64] Tyler S, Hooge MD (2001). Musculature of *Gnathostomula armata* Riedl 1971 and its ecological significance. Mar Ecol.

[65] Leasi F, Todaro MA (2009). Meiofaunal cryptic species revealed by confocal microscopy: the case of *Xenotrichula intermedia* (Gastrotricha). Mar Biol.

[66] Hochberg R, Litvaitis MK (2001). The muscular system of *Dactylopodola baltica* and other macrodasyidan gastrotrichs in a functional and phylogenetic perspective. Zool Scr.

[67] Remane A (1926). Morphologie und verwandtschaftsbeziehungen der aberranten gastrotrichen I. Z Morphol Okol Tiere.

[68] Hochberg R, Litvaitis MK (2001). Functional morphology of muscles in *Tetranchyroderma papii* (Gastrotricha). Zoomorphology.

[69] Remane A (1927). *Xenotrichula velox* nov. gen. nov. spec., ein chaetonotoides Gastrotrich mit männlichen Geschlechtsorganen. Zool Anz.

[70] Hochberg R, Atherton S (2011). A new species of *Lepidodasys* (Gastrotricha, Macrodasyida) from Panama with a description of its peptidergic nervous system using CLSM, anti-FMRFamide and anti-SCPB. Zool Anz.

[71] Rothe BH, Schmidt-Rhaesa A (2008). Variation in the nervous system in three species of the genus *Turbanella* (Gastrotricha, Macrodasyida). Meiofauna Marina.

[72] Remane A (1925). Neue aberrante Gastrotrichen II: *Turbanella cornuta* n. sp. und *T. hyalina* M. Schultze, 1853. Zool Anz.

[73] Teuchert G (1977). The ultrastructure of the marine gastrotrich *Turbanella cornuta* Remane (Macrodasyoidea) and its functional and phylogenetical importance. Zoomorphologie.

[74] Remane A (1927). Beiträge zur Systematik der Süsswassergastrotrichen. Zoologische Jahrbücher (Abteilung für Systematik, Ökologie, und Geographie der Tiere).

[75] Schultze M (1853). Über *Chaetonotus* und *Ichthydium* (Ehrb.) und eine neue verwandte Gattung *Turbanella*. Müller’s Archiv für Anatomie und Physiologie.

[76] Joffe BI, Wikgren M (1995). Immunocytochemical Distribution of 5-Ht (Serotonin) in the Nervous-System of the Gastrotrich *Turbanella cornuta*. Acta Zool.

[77] Schmidt-Rhaesa A, Rothe BH, Schmidt-Rhaesa A, Harzsch S, Purschke G (2016). Gastrotricha. Structure and Evolution of Invertebrate Nervous Systems.

[78] Brusca RC, Brusca GJ (2003). Invertebrates.

[79] Schmidt-Rhaesa A (1996). The nervous system of *Nectonema munidae* and *Gordius aquaticus*, with implications for the ground pattern of the Nematomorpha. Zoomorphology (Berlin).

[80] Schmidt-Rhaesa A (2007). The evolution of organ systems.

[81] Worsaae K, Rouse GW (2008). Is Diurodrilus an annelid?. J Morphol.

[82] Worsaae K, Sterrer W, Iliffe TM (2004). *Longipalpa saltatrix*, a new genus and species of the meiofaunal family Nerillidae (Annelida: Polychaeta) from an anchihaline cave in Bermuda. Proc Biol Soc Wash.

[83] Villora-Moreno S (1996). Ecology and distribution of the Diurodrilidae (Polychaeta), with redescription of *Diurodrilus benazzii*. Cah Biol Mar.

[84] Todaro AM, Perissinotto R, Bownes SJ (2013). Neogosseidae (Gastrotricha, Chaetonotida) from the iSimangaliso Wetland Park, KwaZulu-Natal, South Africa. ZooKeys.

[85] Kisielewksi J (1988). New records of marine Gastrotricha from the French coasts of Manche and Atlantic. 2. Chaetonotida, with descriptions of four new species. Cah Biol Mar.

[86] Kisielewski J (1987). Two new interesting genera of Gastrotricha (Macrodasyida and Chaetonotida) from the Brazilian freshwater psammon. Hydrobiologia.

[87] Mock H (1979). Chaetonotoidea (Gastrotricha) der Nordseeinsel Sylt.

[88] Ehrenberg H (1831). Uber die Entwickelung und Lebensdauer der Infusionsthiere; nebst ferneren Beitragen zu einer Vergleichung ihrer organischen Systeme.

[89] Voigt M (1901). Uber einige bisher unbekannte Siisswasserorganismen. Zool Anz.

[90] Remane A (1951). *Mesodasys*, ein neues Genus der Gastrotricha Macrodasyoidea aus der Kieler Bucht. Kiel Meeresforsch.

[91] Neuhaus B (1987). Ultrastructure of the protonephridia in *Dactylopodola baltica* and *Mesodasys laticaudatus* (Macrodasyda): implications for the ground pattern of the Gastrotricha. Microfauna Marina.

